# The Swine Plasma Metabolome Chronicles "Many Days" Biological Timing and Functions Linked to Growth

**DOI:** 10.1371/journal.pone.0145919

**Published:** 2016-01-06

**Authors:** Timothy G. Bromage, Youssef Idaghdour, Rodrigo S. Lacruz, Thomas D. Crenshaw, Olexandra Ovsiy, Björn Rotter, Klaus Hoffmeier, Friedemann Schrenk

**Affiliations:** 1 Department of Biomaterials & Biomimetics, New York University College of Dentistry, New York, New York, United States of America; 2 Department of Basic Science & Craniofacial Biology, New York University College of Dentistry, New York, New York, United States of America; 3 Department of Palaeoanthropology, Senckenberg Research Institute, Frankfurt am Main, Germany; 4 Department of Biology, New York University Abu Dhabi, Abu Dhabi, United Arab Emirates; 5 Department of Animal Science, University of Wisconsin, Madison, Wisconsin, United States of America; 6 GenXPro GmbH, Frankfurt am Main, Germany; 7 Institute for Ecology, Evolution & Diversity, Goethe University, Frankfurt am Main, Germany; University of Texas Southwestern Medical Center, UNITED STATES

## Abstract

The paradigm of chronobiology is based almost wholly upon the daily biological clock, or circadian rhythm, which has been the focus of intense molecular, cellular, pharmacological, and behavioral, research. However, the circadian rhythm does not explain biological timings related to fundamental aspects of life history such as rates of tissue/organ/body size development and control of the timing of life stages such as gestation length, age at maturity, and lifespan. This suggests that another biological timing mechanism is at work. Here we focus on a "many days" (multidien) chronobiological period first observed as enigmatic recurring growth lines in developing mammalian tooth enamel that is strongly associate with all adult tissue, organ, and body masses as well as life history attributes such as gestation length, age at maturity, weaning, and lifespan, particularly among the well studied primates. Yet, knowledge of the biological factors regulating the patterning of mammalian life, such as the development of body size and life history structure, does not exist. To identify underlying molecular mechanisms we performed metabolome and genome analyses from blood plasma in domestic pigs. We show that blood plasma metabolites and small non-coding RNA (sncRNA) drawn from 33 domestic pigs over a two-week period strongly oscillate on a 5-day multidien rhythm, as does the pig enamel rhythm. Metabolomics and genomics pathway analyses actually reveal two 5-day rhythms, one related to growth in which biological functions include cell proliferation, apoptosis, and transcription regulation/protein synthesis, and another 5-day rhythm related to degradative pathways that follows three days later. Our results provide experimental confirmation of a 5-day multidien rhythm in the domestic pig linking the periodic growth of enamel with oscillations of the metabolome and genome. This association reveals a new class of chronobiological rhythm and a snapshot of the biological bases that regulate mammalian growth, body size, and life history.

## Introduction

### Background to life stages and metabolism-mediated development

The pace and pattern of life stages is the subject of organismal life history, one of biology's most integrative disciplines. The life histories of mammals are described by a number of characteristics, some of which relate to the timing and duration of life stages (e.g., gestation length, age at sexual maturity, lifespan). Body and organ masses are also examined in relation to life history characteristics because body size is fundamental to an organism's physiology and metabolic profile (e.g., adult body mass, neonatal and adult brain weight). What has particularly interested researchers on the life histories of mammals is the covariation that exists between these traits; the characteristics are packaged so tightly to one another within species that no one trait is free to vary without corresponding relative changes in the others [1–4]. But despite this interest, and despite that the physiological underpinnings of each life history trait are well characterized, the factor(s) regulating the combination of traits that define the overall life history matrix of any mammal are *completely* unknown.

Metabolism is responsible for energy allocations that fuel all aspects of life history, modulating the pace and pattern of life [5]. Moreover, as all life history traits reflect dependence on rate and time, metabolic rate must be linked to a biological timing mechanism. At small time scales, the daily biological clock in mammals (and most other organisms) regulates metabolism and apportions energy for the building, functioning, and maintaining of the body. However, while the circadian clock is a key biological mechanism for numerous processes, it has been difficult to link these daily oscillations to the enormous metabolic and life history variation expressed by the entire class of mammals. To address this quandary, we conceptually divide time into two kinds: external time (e.g. daily astronomical variation) that forces circadian time, and biological time (e.g. developmental variation). Discriminating between circadian and biological times permits us to ask the question, 'how can biological time be modified to produce the enormous variation in metabolism-mediated development that we observe'? Because timing is fundamental to establishing all aspects of life history, *we hypothesize that a periodic rhythm longer than the daily biological clock regulates some aspects of metabolic variability that contribute to variability in body size and the pace and pattern of life*.

### Mammalian Enamel Records Evidence of Long Period Rhythms

**Fig 1:**
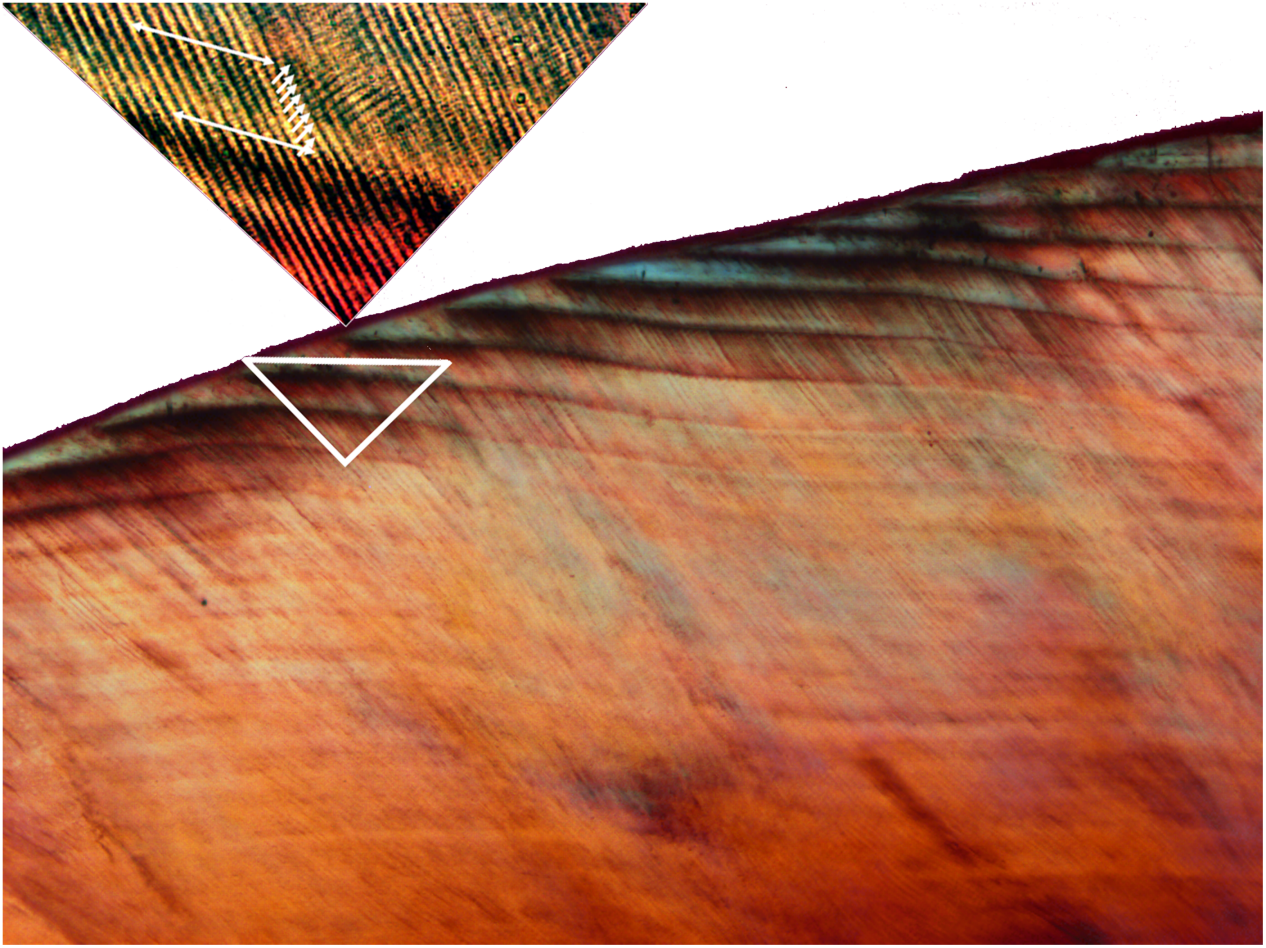
Enamel incremental periodicity. Top: Daily (circadian) growth lines, or cross-striations (short arrows) are observed between adjacent long-period (multidien) striae of Retzius (long arrows) (distance between the adjacent striae of Retzius shown = 30 μm). Bottom: Striae of Retzius may be seen to course across the horizontal field of view (FW = 450 μm). The number of cross-striations between adjacent striae of Retzius is termed the "repeat period" (RP). In this instance the molar enamel from one of the authors (TGB), the RP = 8 (see Fig 2).

**Fig 2:**
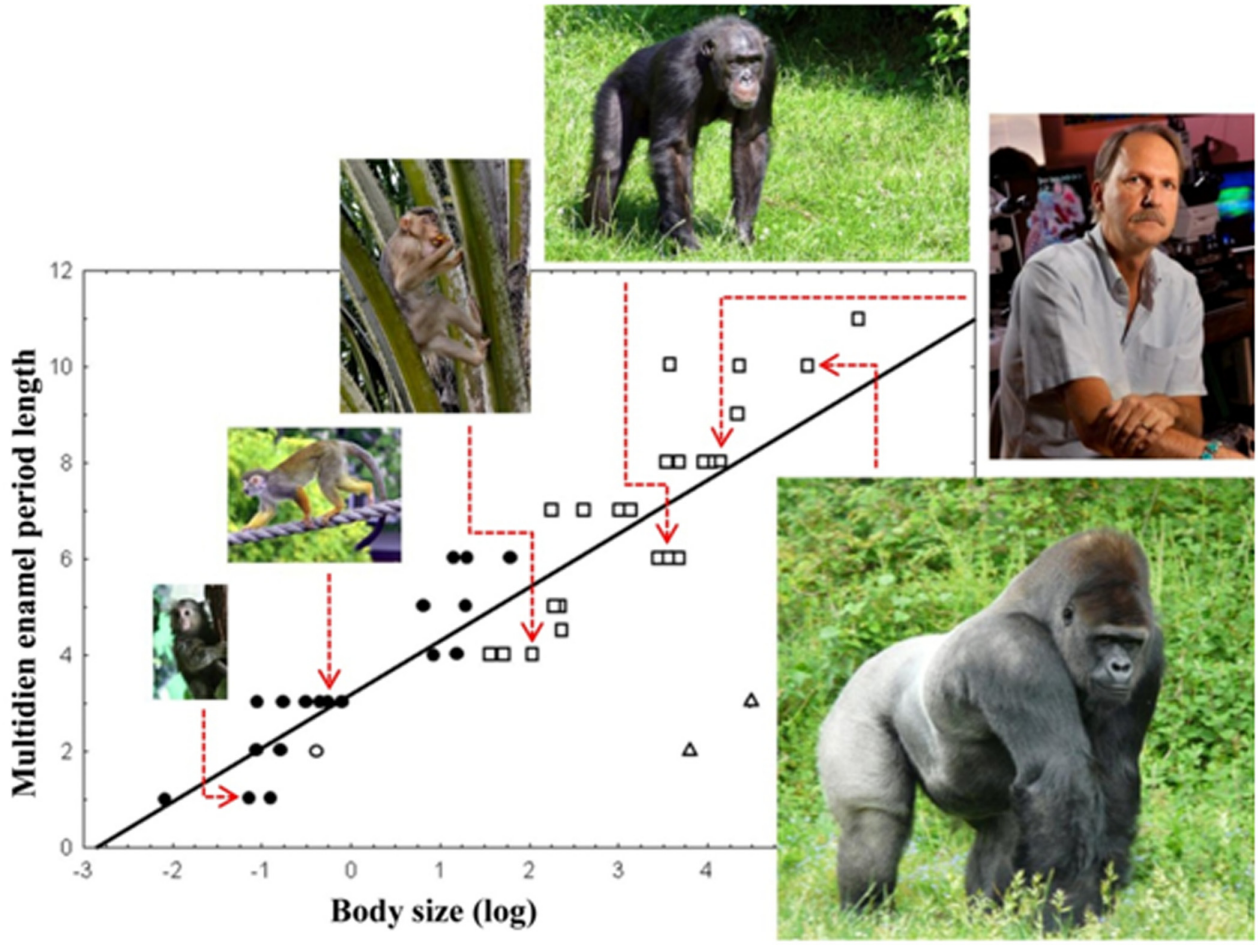
Primate multidien enamel rhythms and body size. Multidien rhythms measured from the enamel of numerous primates are regressed against body size (Kg). This association was our first indication that some systemic physiological event linked to development perturbed the enamel forming cells, altering their matrix production and manifesting a periodic growth line [11]. Lemur outliers have been isolated from other primates on Madagascar for 31 million years, and evolved their limited multidien variability and life history strategy to cope with island ecological instability [12]. Fig 2 is modified from an earlier publication [10]. The following statement is a PLOS requirement: "Tim Bromage pictured in Fig 2 and representing "human", has given written informed consent to himself to publish this image". Closed circles = New World monkeys; Open squares = Old World monkeys, apes; Open triangles = lemurs.

We first encountered long period rhythms from an unlikely place, the skeleton. Mammalian enamel stores in its mineralized microstructure and chemistry a record of responses by its forming cells to systemic circadian and multidien biological rhythms that have long been known to manifest periodic growth lines [6,7] (Fig 1). Whereas the daily growth lines can be explained by the activity of the circadian clock in enamel [8], the long period growth lines, termed striae of Retzius, lack a known chronobiological origin. There is a long standing interest in documenting the number of daily growth lines between adjacent multidien lines among mammals generally, and for non-human primates and early fossil humans specifically [9]. Data accumulated over the past 30 years show that these multidien growth lines have a variation in their repeat period across species. Importantly, small-bodied mammalian species have shorter multidien rhythms than large-bodied species, suggesting a possible metabolic connection with body mass [10]. For example, among primates (from left and clockwise in Fig 2), the tiny marmoset has a rhythm of 1 day, the squirrel monkey's is 3 days, the medium-sized rhesus macaque's is 4-days, the chimpanzee's is 6 days, those of humans average 8–9 days [10] (a third molar extraction revealed Bromage's 8-day rhythm), and the gorilla's rhythm is 10 days. The 11-day data point above the gorilla is a large extinct Asian ape, *Gigantopithecus blacki*, and the outliers (triangles) are insular Madagascar lemurs. Importantly, the range of multidien enamel rhythms significantly correlate highly with all attributes of primate life history [11].

To better understand the link between the enamel multidien rhythm and species' body masses, we found that the enamel rhythm is also highly correlated with all of the body's constituent tissue and organ masses [11,13]. The robust nature of these associations led us previously to term this rhythm the Havers-Halberg oscillation (HHO) [10]. Clearly, the enamel rhythm provides a quantitative measure of developmental time. This biological timing mechanism has been hypothesized to regulate cell proliferation in respect to the accrual of adult body mass and to the emergence of an integrated life history [10,11,13].

A core hypothesis derived from comparative primate enamel research is that multidien rhythms regulate life history by means of centrally controlled body mass-dependent metabolic factors [11]. For instance: gestation length and lactation length are a function of the mass of the mother and are coupled by metabolic rate-dependent energy allocations to offspring [14]; age at sexual maturity depends upon having reached a body mass able to metabolically support a fetus to full term [15]; lifespan is a function of rates of cell proliferation known to regulate longevity [16]. In other words comparing the development of small and large mammal species illustrates that the evolution of large size is the outcome of reducing rates of cell proliferation, but growing at this reduced rate for longer periods of development time (this strategy eventually overcomes the mass "disadvantage" of growing slowly). Thus multidien rhythms, we suggest, must mediate cell proliferation in order to build the required mass in appropriate units of time that then reflect the pace and progression of life stages [11].

We propose that, because all life history traits reflect dependence on rate and time, a biological timing mechanism must be invoked to help explain metabolic regulation of body mass and life history. The circadian clock cannot act as the mechanism by itself, because this timing is common even to mammals with disparate life stages.

Evidently, body size and enamel multidien rhythms share a common biological basis and thus reflect metabolic oscillations beyond the circadian time. To elucidate whether mammals display long period rhythms, we analyzed the metabolomic profile of a medium-sized mammal, the domestic pig, and investigated its enamel rhythm. To our knowledge, this contribution provides the first experimental test in search for mechanisms and a biological basis for the HHO.

## Materials and Methods

Animal care protocols and procedures were reviewed and approved by the University of Wisconsin-Madison Institutional Animal Care and Use Committee (IACUC). The approved protocol number was A01324-0-11-10. Animal were housed in a facility accredited by the Association for the Assessment and Accreditation of Laboratory Animal Care (AAALAC) throughout the experimental procedures. For placement of indwelling catheters pigs were anesthetized for approximately 20 minutes. For anesthesia a mask was placed on the pig snout and they were sedated initially with 7% sevoflurane and maintained to effect with 2 to 4% isoflurane. Pigs were euthanasized prior to tissue collections using procedures described in the 2007 Report of the American Veterinary Medical Association Guidelines on Euthanasia. The pigs were electrically stunned through the brain followed by exsanguination.

Thirty-six crossbred juvenile (pre-estrous) female pigs (gilts) were divided into 2 groups for replicate trials (n = 16 gilts, Trial 1; n = 20 gilts, Trial 2). Gilts were housed individually in pens (2.1 X 0.6 m). Lights were electronically controlled to provide a 12 h light/dark cycle with lights on at 06:00 and off at 18:00 each day. All emergency and stray-light sources were blocked. Red lights that provided <5 lux light were used to facilitate collections during the dark cycle. Three animals acquired an infection during the study period and were excluded, rendering a total of 33 animals for metabolite profiling analysis. Starting at 23:00, a 6 mL blood sample was collected at 2 h intervals via a central venous catheter on days 1, 2, and 14 and once daily on days 3 to 13. On days 3 through 13, blood samples were collected at 23:00 each day. We had to pick a sampling time, and starting at 23:00 and repeating this sampling time for all days of the experiment was chosen assuming that increases in cell proliferation might occur at night.

Two types of mass spectrometry analyses were applied to all samples; gas chromatography-mass spectrometry (GC—MS) and liquid chromatography (LC—MS/MS). To account for inter- and intra-instrumental variation in both GC—MS and LC—MS/MS profiling, data were normalized to the median of reference samples derived from a pool formed from aliquots of all samples. Pooled reference samples were run in parallel through the whole process.

Whereas the amounts of circulating DNA and mRNA were insufficient for genomics of the remaining plasma, it was possible to extract small non-coding RNA (sncRNA) from pooled samples using the exoRNeasy kit (Qiagen), that were then washed, lysed, and separated and loaded onto spin columns. Small RNAs libraries were prepared and 1x50 bps-sequenced on an Illumina HiSeq2000 machine.

All time series data were analyzed using the cosinor method [17–19]; see Supporting Information for more details. Ingenuity Pathway Analysis (IPA; QIAGEN, Redwood City, CA) software was employed for the analysis of metabolites and sncRNA sequences and their respective gene networks. Quantile normalization of sncRNA and metabolites and hierarchical clustering of the correlation matrix were performed using JMP Genomics (SAS Institute, Cary NC, USA). All teeth from the mandibles of each experimental animal were prepared for and imaged by light microscopy to obtain their striae of Retzius multidien rhythm.

All aspects pertaining to the Materials and Methods are elaborated upon in Supporting Information.

## Results and Discussion

**Fig 3:**
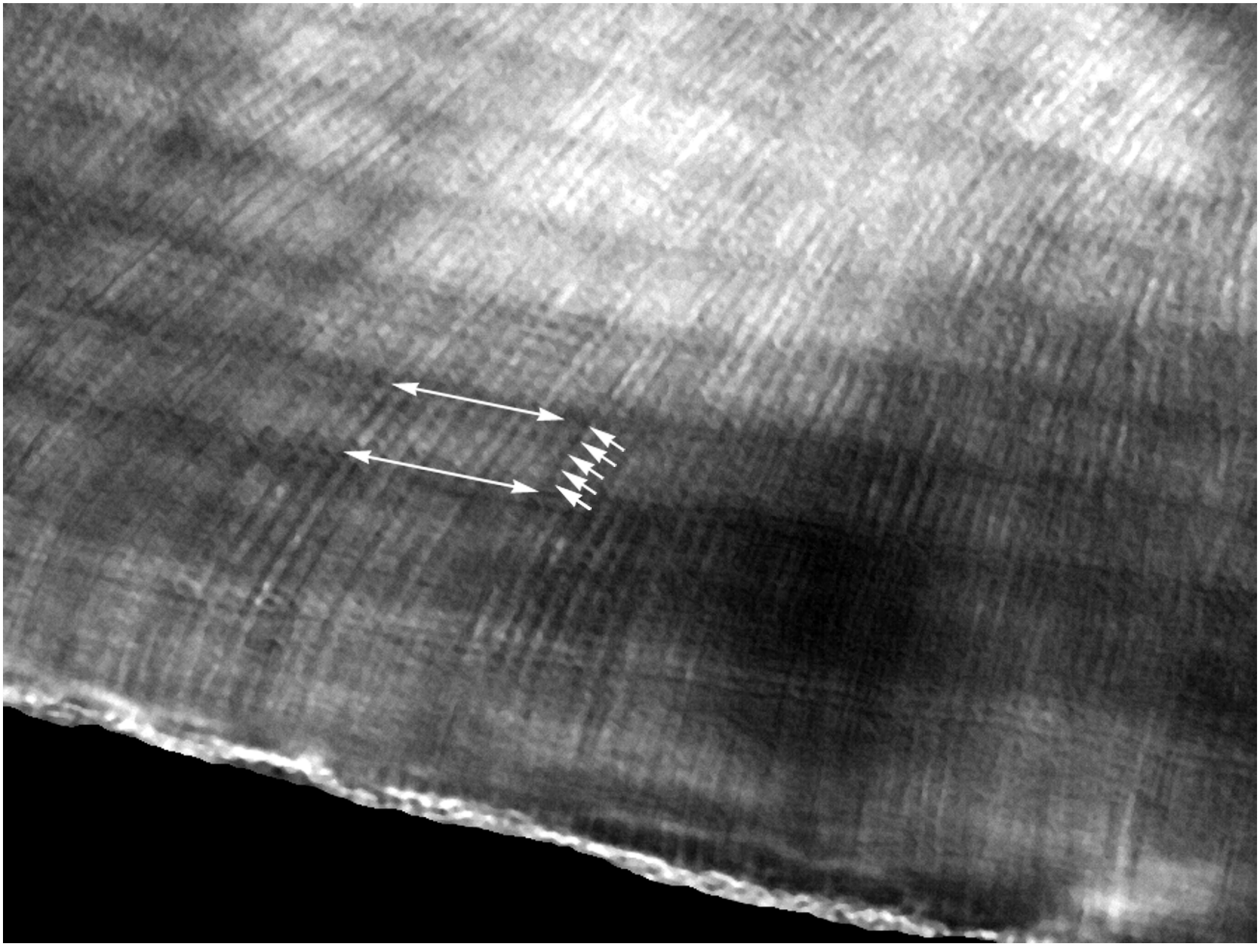
Swine enamel circadian and multidien rhythms. Dark banding across the horizontal field are striae of Retzius (long arrows), while 5 daily events may be seen between adjacent striae (short arrows) (FW = 1 mm).

Teeth from each of our 33 juvenile female farm-raised domestic pigs were surgically extracted for histological analysis to identify individual enamel multidien rhythms; only the periodic value of 5-days was observed (Fig 3) (19 of 33 teeth provided clear striae of Retzius: S1 Table). In addition, we observed teeth that presented accentuated striae of Retzius every other cycle. Together, these periods, we anticipated, would manifest as 5-day and 10-day rhythmic oscillations in the pig metabolome and sncRNAome.

To elucidate biological function of the multidien rhythms, we generated and analyzed 1,551 metabolic profiles from our experimental large mammal sample. These profiles were obtained from blood plasma sampled from each of the 33 pigs daily over a 2-week period. Each sample was subjected to liquid chromatography and gas chromatography analysis coupled with mass spectrometry. Periodicities of circulating metabolites predicted to oscillate on 5-days or 10-days were statistically analyzed using the population-mean cosinor [17,20]. We also obtained blood plasma from the same pigs at 2-hour intervals during the first two days and last day of the procedure to capture 24 hr rhythmicity of metabolites *a priori* known to oscillate on a circadian rhythm. Metabolic analysis was complemented using plasma high-throughput small RNA sequencing.

**Fig 4:**
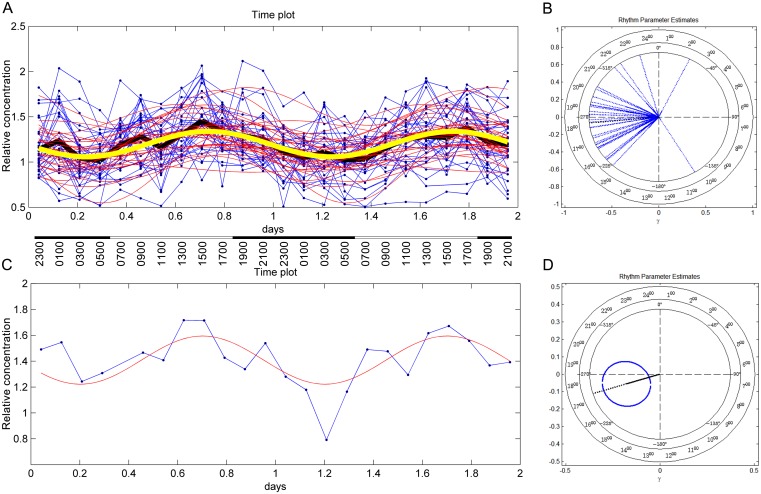
24 hr rhythm of Arginine concentration. A. The cosinor time plot for the metabolite Arginine. Blue points and their connecting lines are original data for each animal in the study; the bold black line is the mean of the data point time series. Red lines are cosinor waves fit over the data for each individual. The bold yellow line is the mean of the individual cosinor time series. On the X-axis, data points are given in decimal fractions of each day and in local time; dark and light bars denote lights-off and lights on illumination conditions. First peak (acrophase) occurs at about 0.75 days (18:00), next peak appearing at 1.75 days on day 2. (All individuals are represented except #12, which was eliminated from the analysis because of having too few measurements for this animal). B. The cosinor polar plot for Fig 4A, illustrating the distribution of first acrophase for each individual, most individuals peaking between 16:00 and 20:00 hr local time. C. The cosinor time plot for a representative individual in the sample from Fig 4A. D. The cosinor polar plot for Fig 4C, illustrating a local time for first acrophase occurring roughly between 17:00 and 18:00 hr.

Spectra for 995 metabolites were identified from the blood samples, from which 228 were classified at high analytical grade (see Supporting Information). Of these, 159 were identified using standards or by elucidating the molecular structure and are of known endogenous and exogenous biological identity, which were examined further. First, we analyzed metabolomic profiles from plasma collected every two hours on days 1–2 and show that the concentrations of 108 metabolites (68%) of known identity, tightly exhibit acrophase and bathyphase suggesting entrainment on a 24 hr rhythm (Fig 4; S2 Table).

Thirty four of 39 amino acids and related metabolite (87%) peaked in the external time of the late afternoon-early evening (15:00–20:00) toward the end of the animals’ active period of the day (S3 Table). Saturated fatty acids also peaked in the the afternoon-early evening (13:00–20:00), while monosaccharides peaked during the late night (01:00–04:00) (S4 Table). These results are highly consistent with metabolome data in diurnal humans [21–23] and with the contrary temporal phasing of metabolome data of a nocturnal species [24], demonstrating the power of using metabolite profiling to capture biological rhythms, and setting the stage for investigating the hitherto enigmatic multidien rhythm.

**Fig 5:**
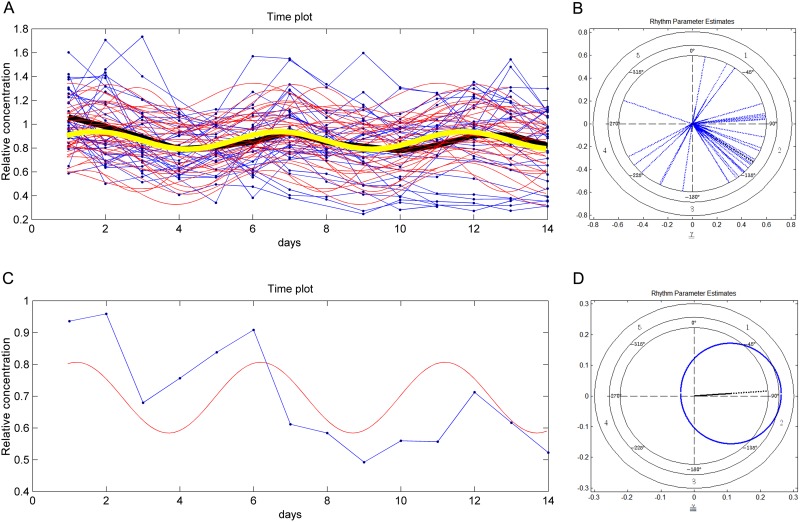
5-day multidien rhythm of Alanine concentration. **A**. The cosinor time plot for the metabolite Alanine. Blue points and their connecting lines are original data for each animal in the study; the bold black line is the mean of the data point time series. Red lines are cosinor waves fit over the data for each individual. The bold yellow line is the mean of the individual cosinor time series. First peak (acrophase) occurs at about day 2, then next peak at day 7, and then day 12. Most metabolites in the study have a more concentrated distribution of first peaks within the day, but this interindividual variability leads to some level of statistical significance for days bordering the rhythm having the highest statistical power. B. The cosinor polar plot for Fig 5A, illustrating the distribution of first acrophase for each individual, most individuals peaking throughout day 2 (see S2 Fig for detail). C. The cosinor time plot for a representative individual in the sample from Fig 5A. D. The cosinor polar plot for Fig 5C, illustrating a local time for first acrophase occurring near to day 2.

To characterize multidien rhythms, we first applied the population-mean cosinor test for all 159 metabolites to the anticipated period length of 5 days. We identified 55 metabolites significantly oscillating on a 5-day period length (range *p* = 0.04–8.13E^-9^; Fig 5, Table 1, S5 Table, S1 Fig). This group of 55 metabolites represents 35% of the 159 metabolites examined.

**Table 1:** Cosinor results for the multidien 5-day "growth" rhythm of the top 10 most significant metabolites from plasma samples collected at 23:00 for days 1–14. See S5 Table for a complete list of metabolites. Mesor is the value about which oscillations occur, Amplitude is half the difference between the highest and the lowest values, and Acrophase is time in degrees at which the first peak of the rhythm occurs. See Supporting Information for more details.

Metabolite	Mesor	Amplitude	Acrophase	p-value	pr%
Stearic acid (C18:0)	0.796 +/-0.062	0.075	-89.600	8.13E-09	21.0
Arachidonic acid (C20:cis[5,8,11,14]4)	0.778 +/-0.056	0.078	-76.500	6.68E-08	23.2
Palmitic acid (C16:0)	0.813 +/-0.067	0.076	-87.500	7.59E-08	19.5
14-Methylhexadecanoic acid	0.935 +/-0.129	0.105	-102.200	1.42E-07	17.4
Citrate	0.779 +/-0.092	0.109	-108.900	4.77E-07	20.1
Heptadecanoic acid (C17:0)	0.921 +/-0.135	0.098	-103.600	1.22E-06	16.1
Phosphate, lipid fraction	0.835 +/-0.054	0.062	-90.600	1.27E-06	17.7
trans-4-Hydroxyproline	0.874 +/-0.087	0.066	-115.400	2.14E-06	13.3
myo-Inositol, lipid fraction	0.806 +/-0.088	0.097	-96.000	2.40E-06	19.0
Asparagine	0.777 +/-0.069	0.102	-116.500	3.04E-06	19.4

While it's investigation is beyond the scope of the present work, we note that the majority of animals are in-phase. The sample is too small to enable a formal statement regarding the nature of synchrony in the population sample, but a tendency among many animals suggests that the timing of breeding and births has an effect on phase position (see Supporting Information for a discussion, S2 Fig).

**Fig 6:**
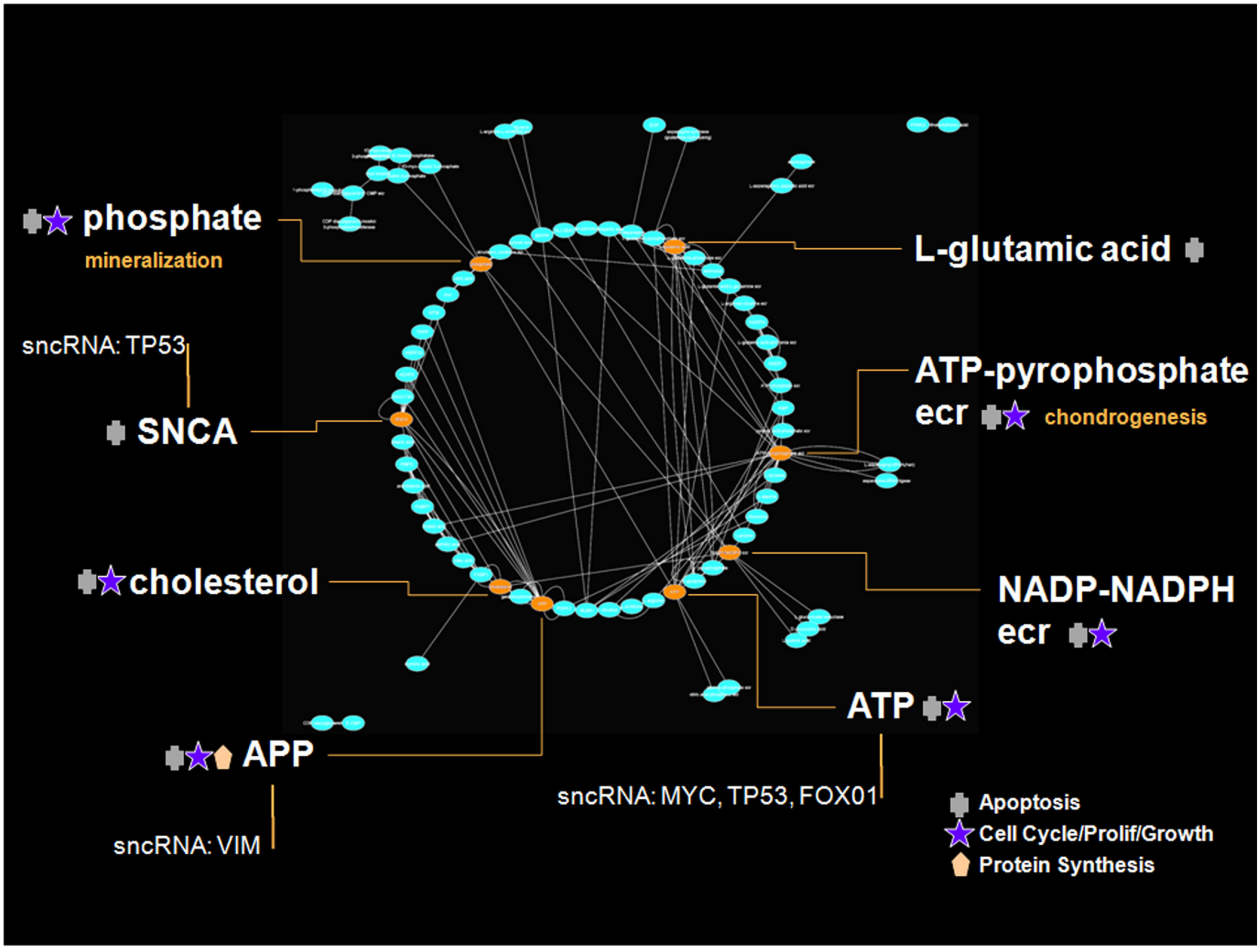
IPA 'direct' associated gene network underlying the 5-day multidien growth rhythm. The directed links for all genes and enzyme-catalysis reactions (ecr) identified in the IPA analysis of the day 2 acrophase metabolite group were entered into Cytoscape [25] to produce this network architecture. Gene hubs containing from 7–17 in- and out-degree links are highlighted, and include ATP-pyrophosphate ecr (17 links), APP (14), L-glutamic acid (11), SNCA (11), ATP (9), phosphate (8), cholesterol (7), and NADP-NADPH ecr (7). Genes included in the sncRNA IPA Gene Interaction Network (TP53, MYC, FOXO1, VIM) are also indicated (see Fig 11).

Intriguingly, the 55 metabolites oscillating on a 5-day period length do not represent one 5-day rhythm, but rather *two* 5-day rhythms separated by 3 days (S6 Table). One sub-group of 45 metabolites has its mean acrophase on the morning of day 2. IPA metabolomics analysis identified three key biological functions of this sub-group, which are regulation of cell proliferation (20 genes), apoptosis (14 genes), and concentration of Ca^2+^ (14 genes) (range *p* = 1.33E^-3^–8.13E^-9^; S7 Table). These primary biological functions are prominent in the gene network, with gene and enzyme-catalysis reactions (ecr) hubs showing high connectivity (Fig 6). (In Fig 6, and the other figures of this sort that follow, we identify primary functions of networks by highlighting those genes with disproportionate connections, or "links", over other genes, which is a reflection of their biological influence. The biological functions of these gene "hubs" are provided by IPA analysis and indicated by symbols in each gene network figure).

**Fig 7:**
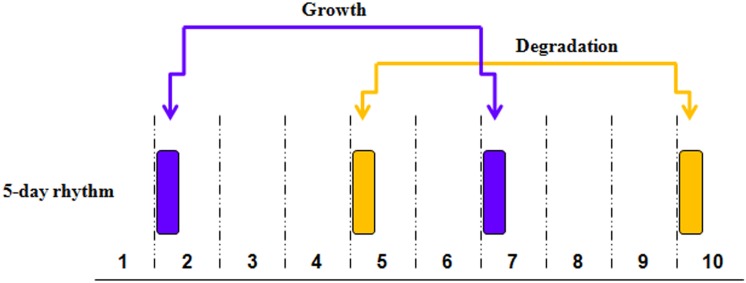
Pattern of 5-day multidien biological functions. The top canonical pathways identified by IPA are: 5-day growth rhythm—Proline Biosynthesis II (from Arginine), tRNA Charging, Citrulline Biosynthesis, Glycine Biosynthesis III, Superpathway of Citrulline Metabolism. 5-day degradation rhythm—Adenine and Adenosine Salvage III, Sucrose Degradation V (Mammalian), Purine Ribonucleosides Degradation to Ribose-1-phosphate, Adenosine Nucleotides Degradation II, Purine Nucleotides Degradation II (Aerobic).

Genes within this network are functionally related to cell cycle/proliferation/growth, apoptosis, and protein synthesis, with additional roles in mineralization and chondrogenesis. The second metabolite group has its mean acrophase 3 days later on the morning of day 5 of the experiment (S6 Table), and has a different biological function than that of the day 2 acrophase group. The metabolites in this group mapped by IPA reveal a dominant degradation and salvage function (Fig 7). This rhythm may represent a tandem response to the metabolic growth events occurring three days earlier, recycling the residuals of those processes to be used again two days later (day 7 from the start of the experiment) in the next cycle of building and maintaining body mass. The in- and out-degree links of the network hubs for both the day 2 and day 5 acrophase groups obey power law distributions, having exponents of 1.45 and 1.52 respectively, consistent with the properties observed in other gene networks [26].

We then tested for the weak but expected 10-day oscillation, revealing a group of 11 metabolites oscillating on this frequency (S8 Table). These metabolites also sort into two rhythmic acrophase groups. One acrophase group is in harmonic-step with the 5-day multidien growth rhythm, converging on day 7 of the experiment (S3 Fig) and sharing the key biological functions of this group, which include cell proliferation and apoptosis. These shared functions occur even though the 10-day acrophase metabolites constitute a completely unique suite of molecules from that of the 5-day growth rhythm (S4 Fig). The second acrophase of the 10-day metabolite group has a dominant degradation function consistent with the biological function of the 5-day degradation group, but which peaks on day 4 of the experiment, one day before the 5-day multidien degradation rhythm.

Even though the biological functions of the 5-day and 10-day metabolite rhythms match, it must be said that with a 14-day record, there is no second repeat of acrophase available for a 10-day periodicity. And while it is accurate to say that even with data not covering a full cycle, the periodicity can still be tested and its parameters estimated for an anticipated frequency. Thus we recommend validation of this weak harmonic signal in future research.

Next we performed high-throughput small RNA sequencing of 3000 sncRNAs that were analyzed for the anticipated multidien rhythms using the cosinor method. We identified 70 sncRNAs that oscillate on a 5-day rhythm (range *p* = 4.94E^-2^–1.01E^-3^; 9 sequences are given in Table 2, and a full list is provided in S9 Table). (The weak 10-day rhythm was not identified). That these 70 sequences have the shortest length in all but one case (S10 Table) suggests that only mature sncRNA are typically oscillatory, and that premature sequences are too transient to be expressed rhythmically; in one case a non-significant mature sncRNA was accompanied by a precursor with a statistically significant 5-day rhythm (TCACCGGGTGTAATCAGCTG).

**Table 2:** Cosinor results are provided for the multidien 5-day "growth" rhythm of the 9 IPA-mapped sncRNA from plasma samples collected at 23:00 for days 1–14. See S9 Table for a complete list of sncRNAs.

sncRNA sequence	Mesor	Amplitude	Acrophase	p-value	pr%
TGGACGTTGGCTCTGGTGGTGA	26.701 +/- 2.515	9.729	-63.293	3.18E-02	46.6
TCAGGCTCAGTCCCCTCCCGATT	264.293 +/- 20.353	76.725	-71.892	3.50E-02	45.6
TCCCTGTCCTCCAGGAGCTC	139.678 +/- 12.228	43.118	-94.209	4.76E-02	42.5
CAAAGAATTCTCCTTTTGGG	433.796 +/- 35.463	144.400	-98.932	2.32E-02	49.6
CTCTAGATAGTCAAGTTCTGATCCAG	41.258 +/- 6.535	23.803	-99.820	4.12E-02	44.0
TGAGGTAGTAGGTTGTGT	31.841 +/- 3.512	14.885	-113.000	1.97E-02	51.0
AATGGCGCCACTAGGGTTGT	34.159 +/- 3.494	12.702	-118.150	4.49E-02	43.1
TTCACCGTGGCTAAGTTCCG	8.388 +/- 1.581	6.310	-157.968	3.61E-02	45.3
CCGGTGAGCTCTCGCTGGCC	32.087 +/- 3.952	16.121	-341.203	3.28E-02	46.3

**Fig 8:**
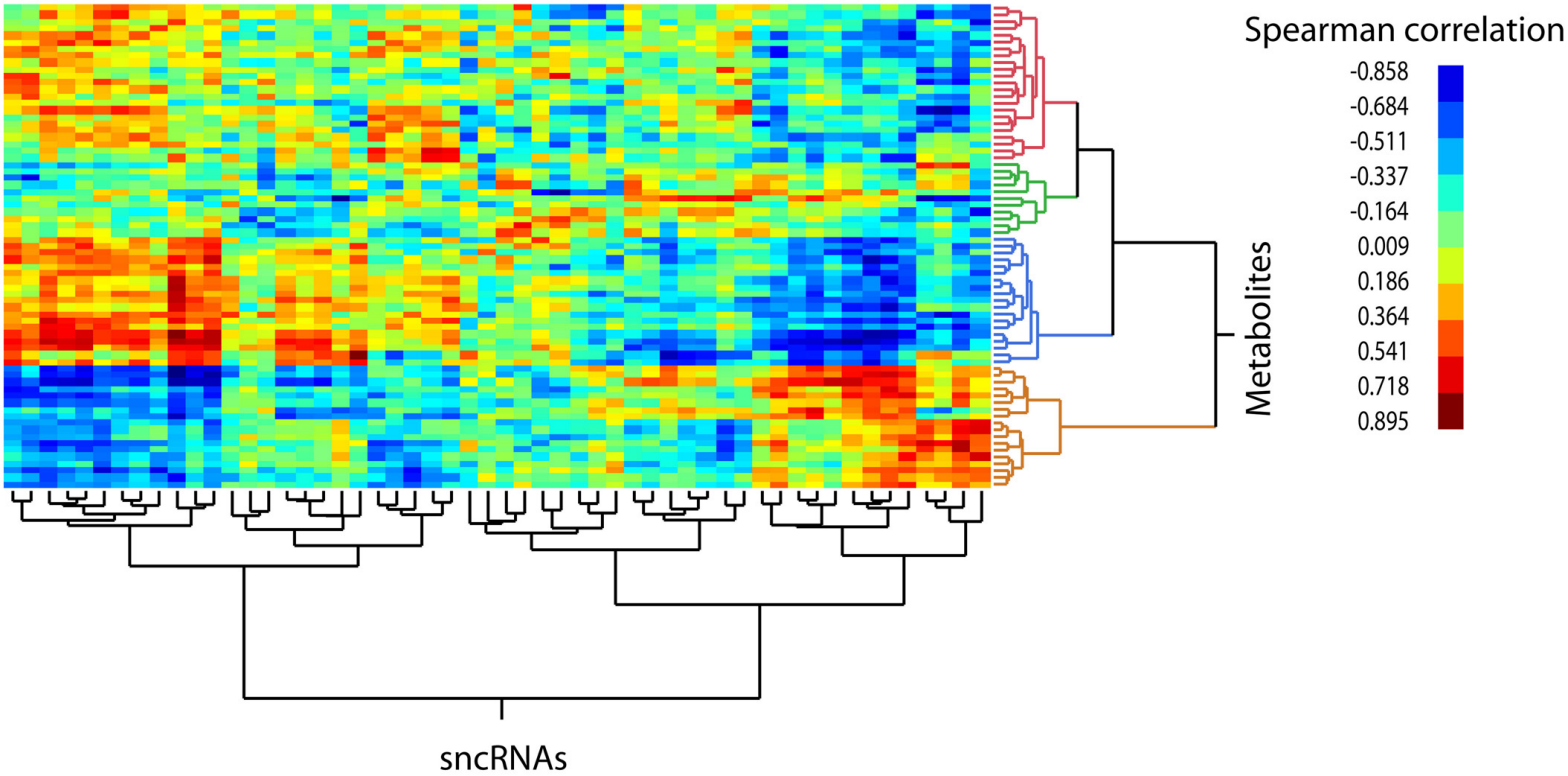
Two-way hierarchical clustering of the cross-correlation matrix of the levels of 70 5-day oscillating sncRNAs and 55 5-day oscillating metabolites across 14 sampling points. Pairwise metabolite-sncRNA Spearman correlations were generated using quantile-normalized metabolite and sncRNA levels. The range of Spearman correlation values is shown to the right of the figure (brown to blue). The sign of correlation values indicate positive (+) and negative (-) correlation. The heat map shows the clustering of metabolites or sncRNAs based on similarity and highlights the presence of major groups of sncRNAs that oscillate in a similar fashion (positive/red-brown or negative/blue) relative to few major groups of metabolites.

We evaluated the consistency of the 5-day rhythm patterns by generating the cross-correlation matrix of all 70 oscillating sncRNAs and 55 oscillating metabolites and grouped these correlations into broad clusters. This analysis revealed strong cross-correlation structure in the data such that subsets of each dataset show consistent patterns of variation across the 14 sampled time points (Fig 8), validating the underlying molecular mechanisms responsible for the existence of the multidien rhythm.

**Fig 9:**
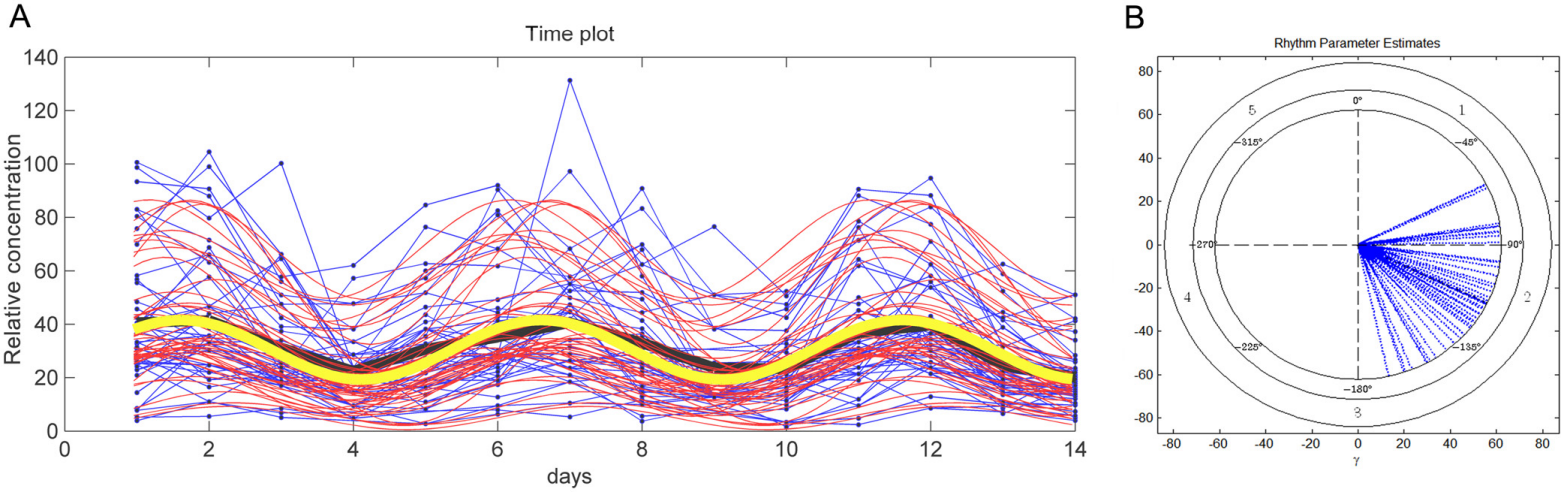
Multidien rhythm of sncRNA concentration. **A**. The cosinor time plot for 44 of 52 sncRNA representing the growth rhythm (eight very high concentration sncRNAs were removed to expand the scale for visualizing the mean cosinor rhythm). Blue points and their connecting lines are original data for each pooled sncRNA in the study; the bold black line is the mean of the data point time series. Red lines are cosinor waves fit over the data for each pooled sncRNA. The bold yellow line is the mean of the individual cosinor time series. First peak (acrophase) occurs at about day 2, then next peak at day 7, and then day 12. Lower right. All sncRNA in the study have a concentrated distribution of first peaks within day 2. B. The cosinor polar plot for Fig 9A, illustrating the distribution of first acrophase for each individual, most individuals peaking throughout day 2.

**Fig 10:**
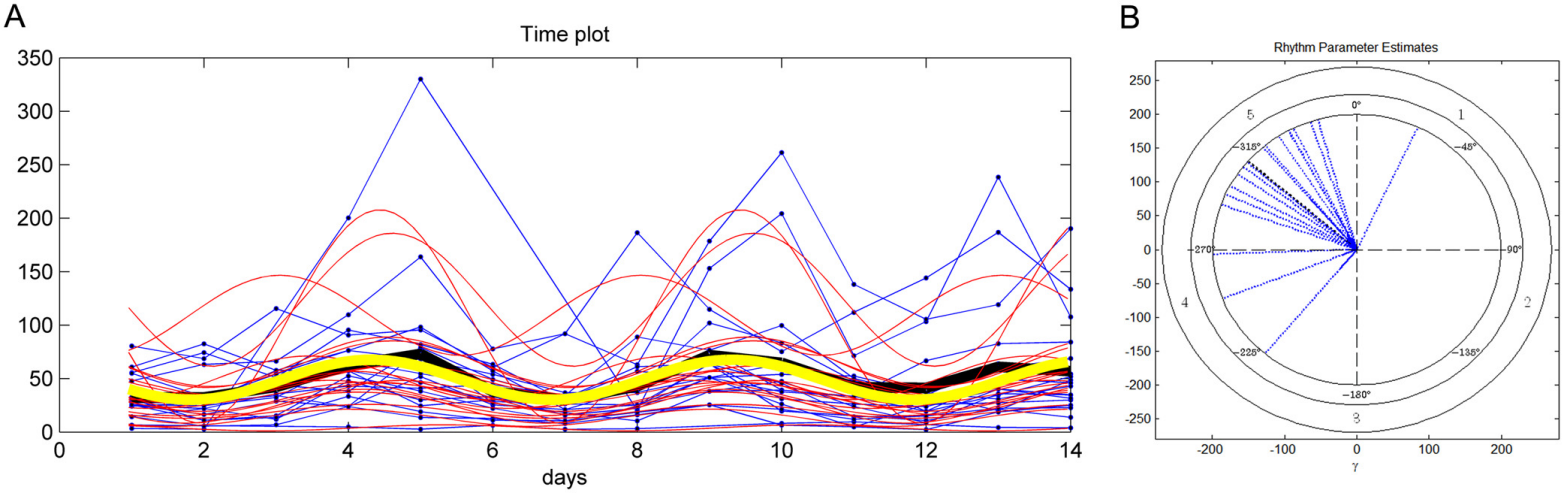
Multidien rhythm of sncRNA concentration. **A**. The cosinor time plot for 18 of 19 sncRNA representing the degradation rhythm (one very high concentration sncRNA was removed to expand the scale for visualizing the mean cosinor rhythm). Blue points and their connecting lines are original data for each pooled sncRNA in the study; the bold black line is the mean of the data point time series. Red lines are cosinor waves fit over the data for each pooled sncRNA. The bold yellow line is the mean of the individual cosinor time series. First peak (acrophase) occurs at about early day 5, then next peak at early day 10. Most sncRNA in the study have a concentrated distribution of first peaks within day 5. B. The cosinor polar plot for Fig 10A, illustrating the distribution of first acrophase for each individual, most individuals peaking during day 5.

**Fig 11:**
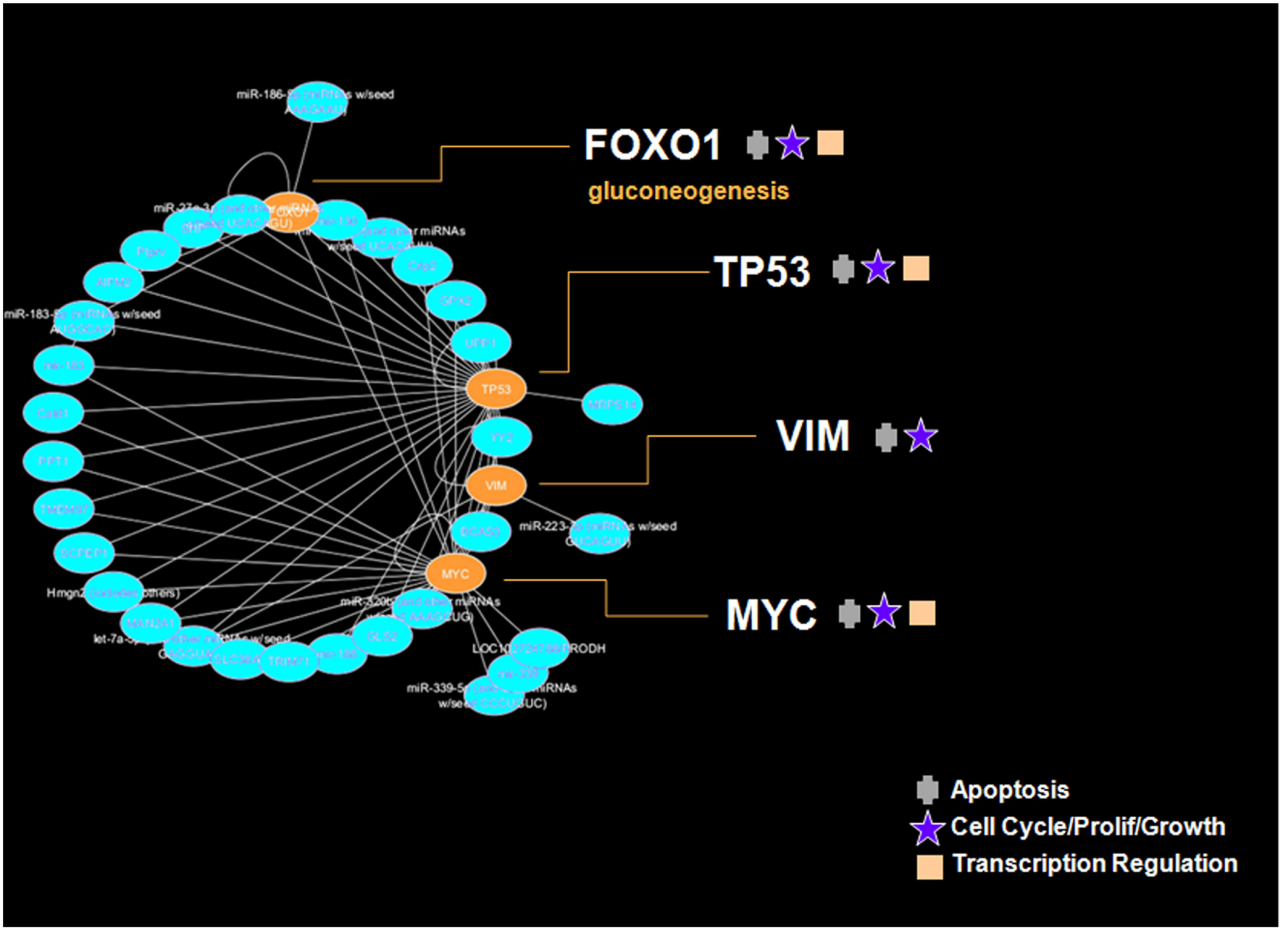
IPA 'direct' associated gene network underlying the 5-day multidien growth rhythm. The directed links for all genes identified in the IPA analysis of the day 2 acrophase sncRNA group were entered into Cytoscape [25] to produce this network architecture. Gene hubs containing from 7–27 in- and out-degree links are highlighted, and include TP53 (27 links), MYC (23), VIM (8), and FOXO1 (7). Genes underlying the 5-day and10-day metabolite multidien growth rhythms are included in the sncRNA IPA Gene Interaction Networks illustrated in Fig 8 and S4 Fig respectively.

The strong cross-correlation pattern is apparent also when all 422 annotated sncRNAs and 55 oscillating metabolites are subject to this analysis (S5 Fig). The sncRNAs sort into acrophase mean expression peaks on days 2 and 5 (Figs 9 and 10 respectively). Evaluation of the day 2 sncRNAs by IPA analysis generates a gene network that regulates the same biological functions described by the gene network connected with the day 2 metabolites; namely, cell proliferation, apoptosis, and transcription regulation/protein synthesis (Fig 11). While the day 2 sncRNAs represent a small proportion of all molecules evaluated, the probability that the results have been obtained by chance alone is, we think, so astronomically small as to be quite impossible. Given that 1) the sncRNA follow the two 5-day rhythms consistent with those of the metabolome, that 2) the sncRNA and metabolites share the same biological functions, that 3) 69 of 70 sncRNA oscillate in their mature form and not their many precursors, and that 4) the cross correlation matrix is so strong between sncRNA and metabolite concentrations, gives us every confidence that our spectral analysis has identified the multidien rhythm known a priori from the enamel.

The day 5 sncRNA acrophase group contains one pig-specific [27] sequence (S9 Table) and one sequence mapped by IPA and annotated to the human genome as mir-4792. This sncRNA acts directly on mir-344a-5p, which has its principal function to inhibit adipocyte differentiation [28]. The extent to which the day 5 sncRNAs follows the degradative and salvage functions of the late acrophase group of metabolites requires further study, but nonetheless the biological function of mir-4792 is anti-proliferative, and thus, consistent with day 5 metabolites, is contrary to the function of day 2 sncRNAs.

## Conclusions

Extensive molecular, cell, and behavioral research has documented the regulation of daily cycles by the circadian clock, coordinating metabolic activities to appropriate times of the 24 h solar day. That this biological rhythm dominates the chronobiology paradigm agrees with our expectations because cell proliferation research is largely performed in the mouse, which is a "1-day" sized mammal. In addition, cell cycle studies in humans are undertaken on bone marrow, gut, oral mucosa, and cultured fibroblasts, which reflect tissue-specific dynamics of cell replacement and colonization [29], *not* growth and development. Such research foci cannot detect multidien physiological rhythms regulating growth. To overcome this barrier we have undertaken our chronobiological research on a large-bodied mammal.

The production and maintenance of tissue/organ/body mass is a function of the long established dependence of the cell cycle upon the daily biological clock [30–39]. Cell proliferation is metabolically expensive, which underscores the observation that small and fast growing mammals have high tissue-specific metabolic rates relative to those of large and slower growing mammals; small mammals have only the circadian rhythm to build their mass. The rationale behind our study is that this difference between small and large mammals signifies the existence of an additional biological timing mechanism unique to the development of slow growing large-bodied mammals, which works by slowing down the rate of cell proliferation over a multiple of days.

Data shown here demonstrate that the domestic swine metabolome chronicles a 5-day metabolic rhythm, bolstered every other cycle, we suggest, by a minor 10-day rhythm. These rhythms are linked to pace of cell proliferation, the concomitant cell death that accompanies growth and development, and transcription regulation and protein synthesis. In addition, the metabolite rhythm is coupled to the concentration of Ca^2+^, which is known to have regulatory roles in apoptosis, transcription, and enzyme and protein conformation involved in growth [40]. Overall, the biological functions highlighted by IPA converge upon the metabolic requirements of growth [41] and are important mediators of the pace at which body mass is achieved [42].

We might not have reached this stage in our research if it had not been for the recording of multidien rhythms in mammalian enamel. Enamel forming cells are clearly sensitive to the systemic physiological manifestation of the multidien rhythm, and they mineralize this signal (perturbation?) into its secreted matrix as a stria of Retzius. Therein is an irony, in that striae of Retzius have been a focus of research on enamel structure for more than a century, yet these striae have nothing particularly to do with enamel as a biomaterial. What is wonderful, however, is that enamel is a window into life history that, with the present molecular evidence advanced, will allow us to reconstruct the evolutionary biology of taxa, such as for the Order Primates, with greater depth and into the paleontological record.

To our knowledge, this study provides the first experimental evidence for a multidien rhythm that may be important in regulating the pace of development and engendering a life history. Till now an explanation for the patterning of mammalian life has remained enigmatic [2,3]. Our previous studies have suggested that the rate at which adult body mass is achieved is key to what regulates species life history evolution, by enforcing mass-dependent chronobiological restrictions over the timing of life stages [11,13]. Interspecifically, rates of production—i.e., body mass increase—are slow when the HHO is long, which leads to longer periods of growth and larger final adult body mass. Our experimental findings on the domestic pig metabolome, which highlight biological functions related to the mediation of growth and development, are in keeping with this notion.

Although our study identified changes in metabolomic signatures with a principal 5-day period, the upstream biological regulators enhancing key functions related to growth remain obscure. We do not claim that all mammalian taxa have followed this strategy, nor even all representatives within major taxonomic groupings, but our data, however, enables us to frame a working hypothesis for interpreting why some species present differences in HHO.

Mammals generally, and primates specifically, originated from small-bodied organisms that adapted to external time (daily astronomical rhythms) by regulating biological time to the same period length (circadian rhythms) [11]. Natural selection favoring larger body mass would favor mutations that produced longer growth rhythms and slower development in deference to the constraint imposed by circadian rhythms, that constraint being the metabolic challenge of increasing tissue specific metabolic rates at larger body sizes in a finite energetic model. While circadian oscillators remained the indubitable adaptation to the astronomical cycle of our world, selection pressures favoring larger body mass would thus have to operate on this constraint that circadian rhythms place on growth. HHO multidien rhythms, we suggest, evolved in response to these pressures among some taxa, generating diversity in body mass and hence of life history. Our study suggests that oscillations of the metabolome and sncRNAome, regulated by some as yet to be identified, but presumed central biological timing mechanism, have enabled the diversification of multidien rhythms and body mass for some mammalian taxa. To build upon this working hypothesis it will be important to repeat the results in another species in which the periodicity of the enamel growth lines is significantly different from that in pigs. In the meantime, we suggest that the domestic pig's HHO cell proliferation rhythms must have lengthened in respect to those of its smaller ungulate ancestors, leading to slower and longer periods of growth and larger final adult body mass. We conclude that multidien biological timing has been co-opted among some mammalian taxa to generate HHO rhythms, which help regulate the rate at which body mass is built and, through this mechanism, their life histories.

## Supporting Information

S1 Fig Multidien rhythm of metabolite concentrations. Here are represented the cosinor time plots for a variety of metabolites. Points, but not lines are original data for each animal in the study; the bold black line is the mean of the data point time series. Gray lines are cosinor waves fit over the data for each individual, from which the statistics derive; the bold orange line is the mean of the cosinor time series. The left-side panel represent day2 metabolites, in which first peak (acrophase) is at about day 2, then next acrophase at about day 7, and then day 12. The right-side panel represent day5 metabolites, in which first peak (acrophase) is at about day 5, then next acrophase at about day 10.
(TIF)

S2 Fig Distribution of acrophase for the metabolite Alanine. Here is represented the polar plot of a population-cosinor analysis for the metabolite Alanine (see Fig 5, main text). Most animals are in phase around day 2 (see section **Animal metabolite synchrony** in Supporting Information).
(TIF)

S3 Fig Functional pattern of 5- and 10-day multidien biological functions. The top canonical pathways identified by IPA are: **5-day growth rhythm:** Proline Biosynthesis II (from Arginine), tRNA Charging, Citrulline Biosynthesis, Glycine Biosynthesis III, Superpathway of Citrulline Metabolism. **5-day degradation rhythm:** Adenine and Adenosine Salvage III, Sucrose Degradation V (Mammalian), Purine Ribonucleosides Degradation to Ribose-1-phosphate, Purine Ribonucleosides Degradation to Ribose-1-phosphate. **10-day growth rhythm:** NAD Biosynthesis III, Ceramide Degradation, Sphingosine and Sphingosine-1-phosphate Metabolism, Phosphatidylethanolamine Biosynthesis II, tRNA Splicing; **10-day degradation rhythm:** Urate Biosynthesis/Inosine 5'-phosphate Degradation, Arginine Degradation I (Arginase Pathway), Arginine Degradation VI (Arginase 2 Pathway), Guanosine Nucleotides Degradation III, Adenosine Nucleotides Degradation II.
(TIF)

S4 Fig IPA 'direct' associated gene network underlying the 10-day multidien growth rhythm. The directed links for all genes and enzyme-catalysis reactions (ecr) identified in the IPA analysis of late acrophase metabolites were entered into Cytoscape [25] to produce this network architecture. Gene hubs containing from 3–5 in- and out-degree links are highlighted. Genes included in the sncRNA IPA Gene Interaction Network (TP53, MYC) are also indicated (see Fig 11, main text).
(TIF)

S5 Fig Two-way hierarchical clustering of the complete cross-correlation matrix of the levels of annotated sncRNAs (n = 442) and 5-day oscillating metabolites (n = 56) across 14 sampling points. This heat map is similar to the heat map shown in Fig 9 but included all annotated 442 sncRNA and not just the 5-day oscillating sncRNAs. Pairwise metabolite-sncRNA Spearman correlations were generated using quantile-normalized metabolite and sncRNA levels. The range of Spearman correlation values is shown to the right of the figure (brown to blue). The sign of correlation values indicate positive (+) and negative (-) correlation. Clustering within each group (Metabolites or sncRNAs) is based on similarity.
(TIF)

S6 Fig Cross-correlation of 422 annotated sncRNAs and 56 5-day oscillating metabolites across 14 sampling points. Distribution of 23,632 Spearman correlations corresponding to 422 sncRNA x 56 metabolites (A). Examples of positive and negative sncRNA-metabolite correlations observed in cross-correlation analysis (B). The cross-correlation matrix in S5 Fig is a rich source of metabolome-genome relationships when tests are performed on multidien timescales. For instance, here, the identification of sncRNA mir 455-5p known to inhibit colorectal cancer proliferation and invasion [43] is correlated with free cholesterol levels, and may warrant pharmaceutical research.
(TIF)

S1 Materials and Methods Details are provided that address the following points: 1. Animal number; 2. Animals, care, housing, and diets; 3. Indwelling venous catheter placement; 4. Blood collections and plasma separation; 5. Tooth collection, histology, and imaging; 6. Metaboloite profiling; 7. Animal metabolite synchrony; 8. Isolation of plasma small non-coding RNA (sncRNA); 9. Preparation of small RNA-sequencing (sRNA-seq) libraries; 10. Oscillation statistical analysis; 11. Cross-correlation analysis; 12. References.
(PDF)

S1 Table Animal husbandry and study details. (XLSX)

S2 Table Metabolites of high analytical grade. Cosinor results are provided for metabolites having circadian rhythms for days 1–2.
(XLSX)

S3 Table Cosinor results are provided for circadian rhythms of amino acid and related metabolite concentrations for days 1–2. (XLSX)

S4 Table Cosinor results are provided for circadian rhythms of metabolites other than amino acids and related in respect of metabolite concentrations for days 1–2. (XLSX)

S5 Table Cosinor results are provided for a multidien 5- and 10-day rhythms of plasma metabolites from 23:00. (XLSX)

S6 Table Cosinor results are provided for a multidien 5-day rhythm of plasma metabolites from 23:00 plasma samples for days 1–14. (XLSX)

S7 Table Biological functions of the day 2 acrophase group with a 5-day multidien oscillation of plasma metabolites identified from a direct IPA analysis. (XLSX)

S8 Table Cosinor results are provided for a multidien 10-day rhythm of plasma metabolites from 23:00 plasma samples for days 1–14. (XLSX)

S9 Table Cosinor results are provided for the day 2 acrophase group with a multidien 5-day rhythm of plasma sncRNA from 23:00 plasma samples for days 1–14. (XLSX)

S10 Table Plasma 5-day rhythm sncRNA sequences (check marks) together with families of related sequences identified in this study. (XLSX)

S11 Table Raw concentration data are provided for plasma metabolites following a multidien 5-day rhythm (see S6 Table; non-highlighted are day 2 acrophase group and highlighted are day 5 acrophase group). (XLSX)

S12 Table Raw concentration data are provided for plasma sncRNA following a multidien 5-day rhythm (see S9 Table). (XLSX)
