# Correction: The Swine Plasma Metabolome Chronicles "Many Days" Biological Timing and Functions Linked to Growth

**DOI:** 10.1371/journal.pone.0301870

**Published:** 2024-04-02

**Authors:** Timothy G. Bromage, Youssef Idaghdour, Rodrigo S. Lacruz, Thomas D. Crenshaw, Olexandra Ovsiy, Björn Rotter, Klaus Hoffmeier, Friedemann Schrenk

The original image presented in Fig 3 exhibited growth increments formed during a 24 hour daily cycle as opposed to the intended figure demonstrating long period growth lines exhibited in enamel every 5 days. This error was caused by a mistake during assembly of the figures.

**Fig 3:**
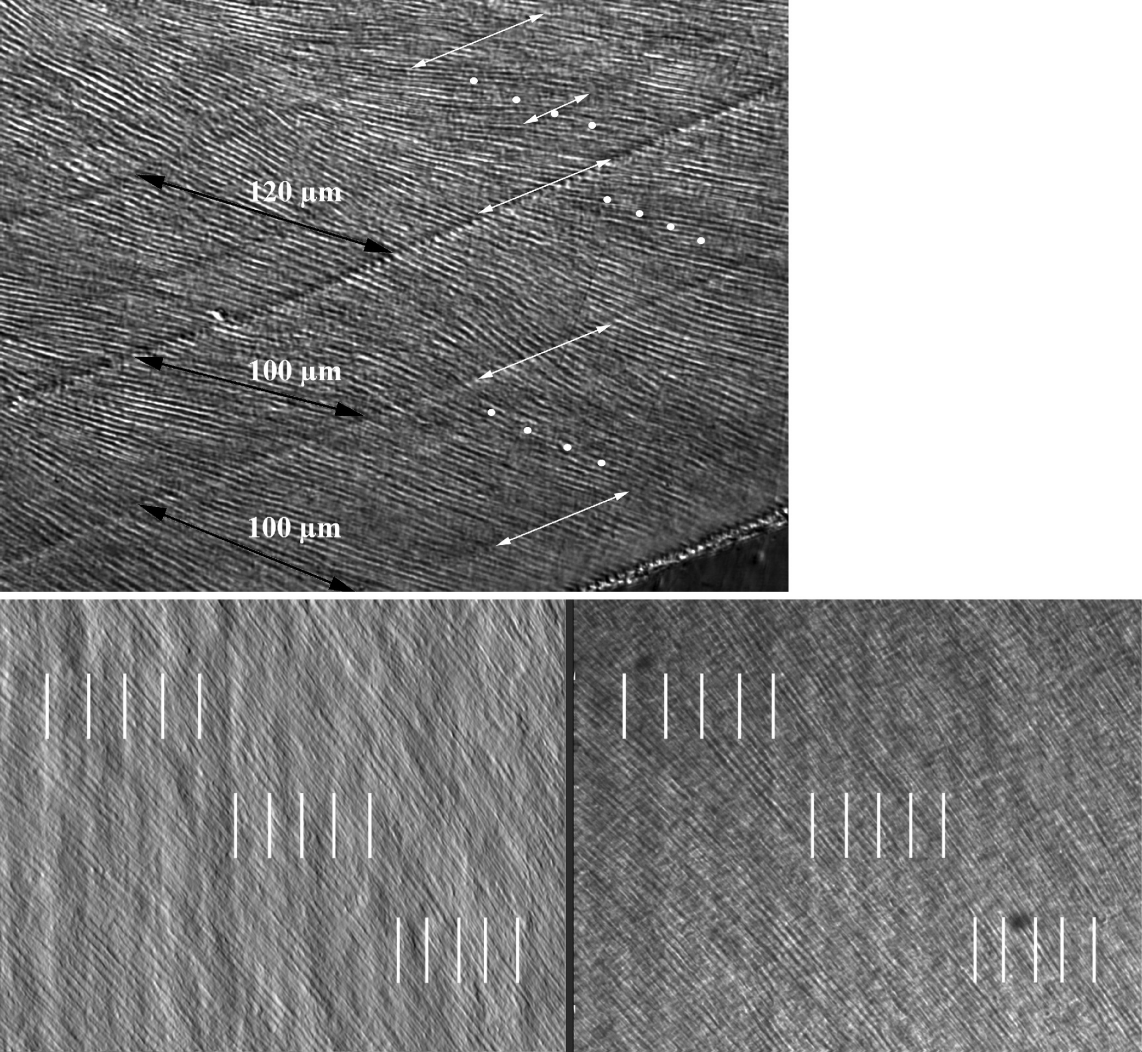
Long-period growth lines. Top: Differential interference contrast (DIC) image of cervical lateral enamel from a first permanent mandibular molar. Multidien growth lines (long white arrows) and daily growth lines (white dots) recorded from through-focusing. Distances parallel with the enamel prisms are measured between three consecutive sets of multidien growth lines (black arrows). One accentuated growth line on a day 3 is illustrated (short white arrow), possibly representing the 5-day degradation rhythm (see Fig 7). Image acquired with a Leica DM5000 B using a 20x HC PL Fluotar NA 0.5 objective lens. FW (width of field) = 358 µm. Bottom: Profilometry of the first surface of a histological thin section of mid-crown-height lateral enamel from a first permanent mandibular molar; profilometry was performed using Get Phase interferometry (v. 3.3.4.0, PhaseView, Buisson, France). Left: simulated DIC. Right: an incident light image of the same field of view reveal daily increments of about 13–18 µm apart (white lines). Image acquired with a Zeiss AxioImager M1m using a 20x Epiplan HD NA 0.4 objective lens. FW = 490 µm each image.

With this correction the authors provide a corrected version of Fig 3 with a replacement image from the original experiments and an updated figure legend. All underlying data can be found in the supporting information files associated with this article.

The authors apologize for the error in the published article.
