# Supplementary figures and images for: The Swine Plasma Metabolome Chronicles "Many Days" Biological Timing and Functions Linked to Growth
Source: PLoS One. 2016 Jan 6;11(1):e0145919. doi: 10.1371/journal.pone.0145919 (PMC4703299; doi:10.1371/journal.pone.0145919)

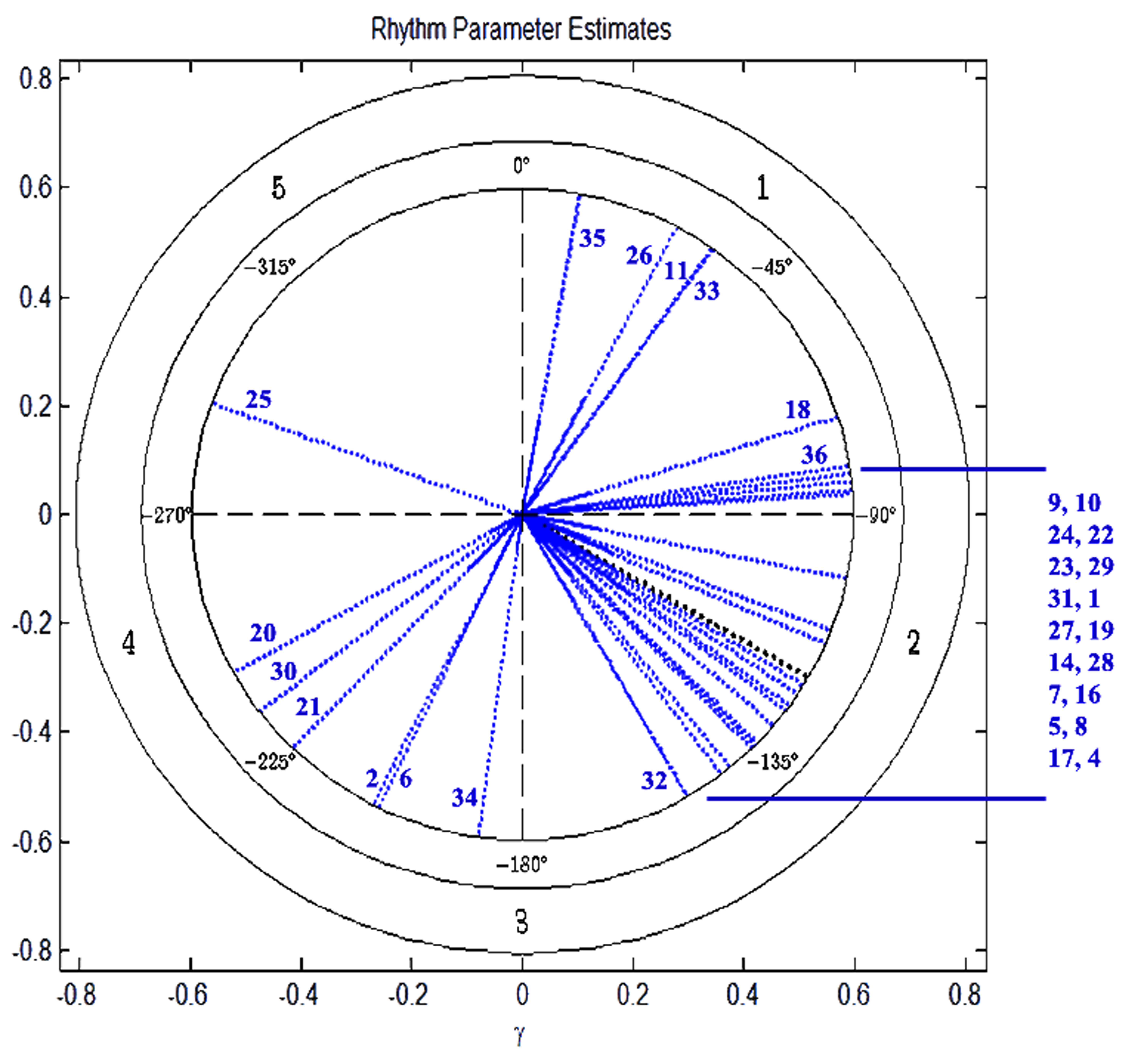

Supplement: S2 Fig — Here is represented the polar plot of a population-cosinor analysis for the metabolite Alanine (see Fig 5, main text). Most animals are in phase around day 2 (see section Animal metabolite synchrony in Supporting Information). (TIF) [file pone.0145919.s002.tif]

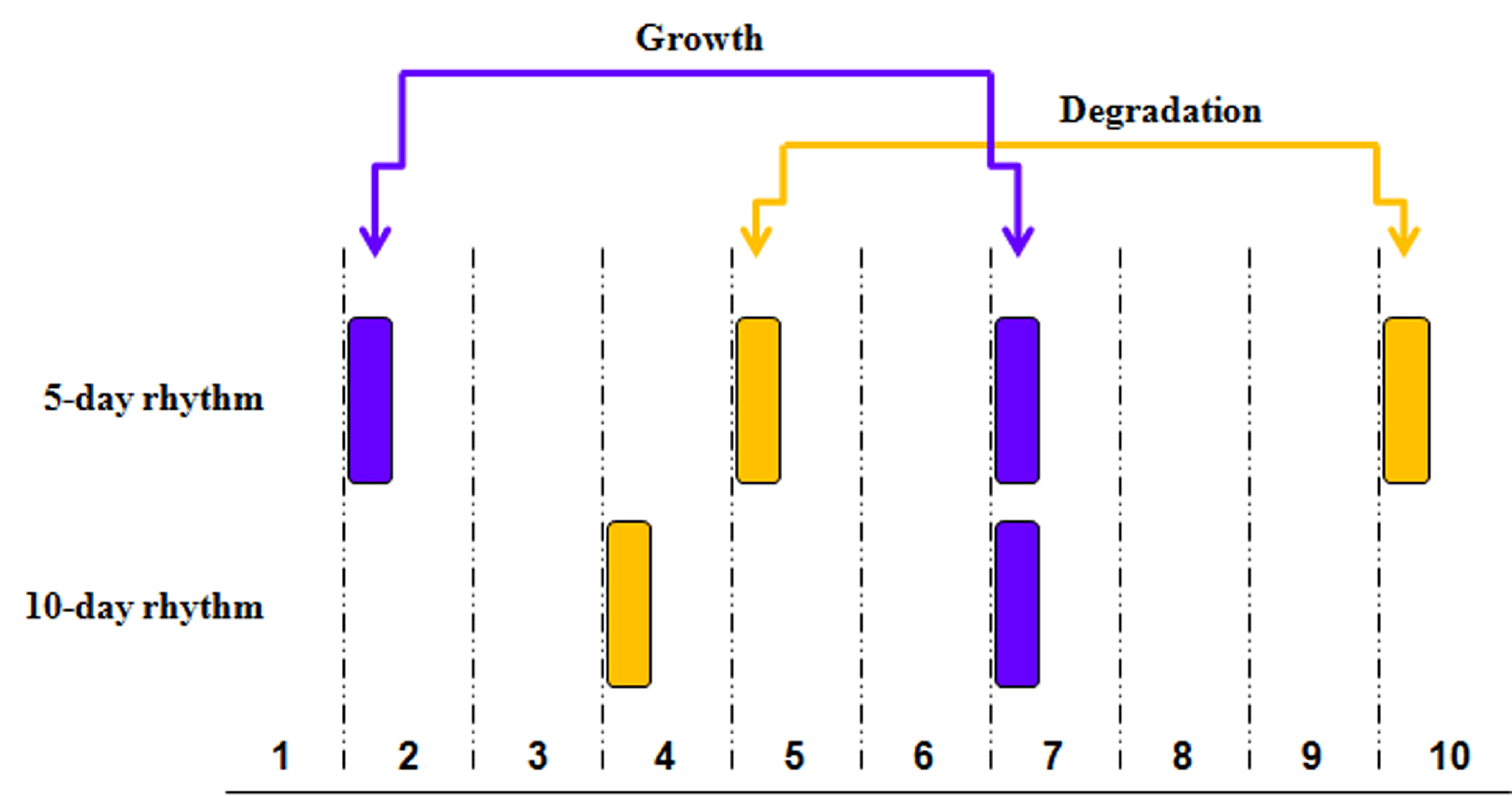

Supplement: S3 Fig — The top canonical pathways identified by IPA are: 5-day growth rhythm: Proline Biosynthesis II (from Arginine), tRNA Charging, Citrulline Biosynthesis, Glycine Biosynthesis III, Superpathway of Citrulline Metabolism. 5-day degradation rhythm: Adenine and Adenosine Salvage III, Sucrose Degradation V (Mammalian), Purine Ribonucleosides Degradation to Ribose-1-phosphate, Purine Ribonucleosides Degradation to Ribose-1-phosphate. 10-day growth rhythm: NAD Biosynthesis III, Ceramide Degradation, Sphingosine and Sphingosine-1-phosphate Metabolism, Phosphatidylethanolamine Biosynthesis II, tRNA Splicing; 10-day degradation rhythm: Urate Biosynthesis/Inosine 5'-phosphate Degradation, Arginine Degradation I (Arginase Pathway), Arginine Degradation VI (Arginase 2 Pathway), Guanosine Nucleotides Degradation III, Adenosine Nucleotides Degradation II. (TIF) [file pone.0145919.s003.tif]

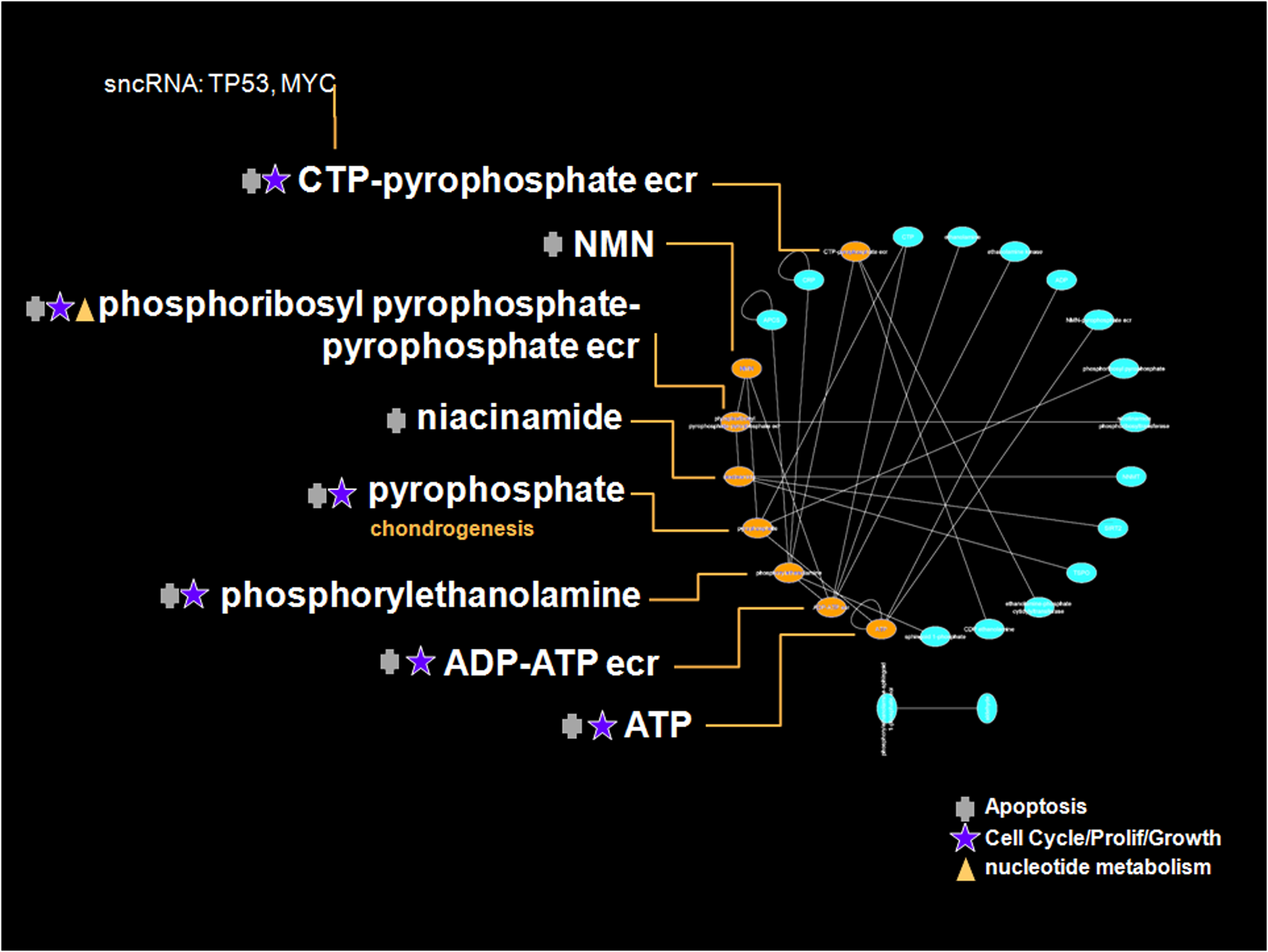

Supplement: S4 Fig — The directed links for all genes and enzyme-catalysis reactions (ecr) identified in the IPA analysis of late acrophase metabolites were entered into Cytoscape [25] to produce this network architecture. Gene hubs containing from 3–5 in- and out-degree links are highlighted. Genes included in the sncRNA IPA Gene Interaction Network (TP53, MYC) are also indicated (see Fig 11, main text). (TIF) [file pone.0145919.s004.tif]

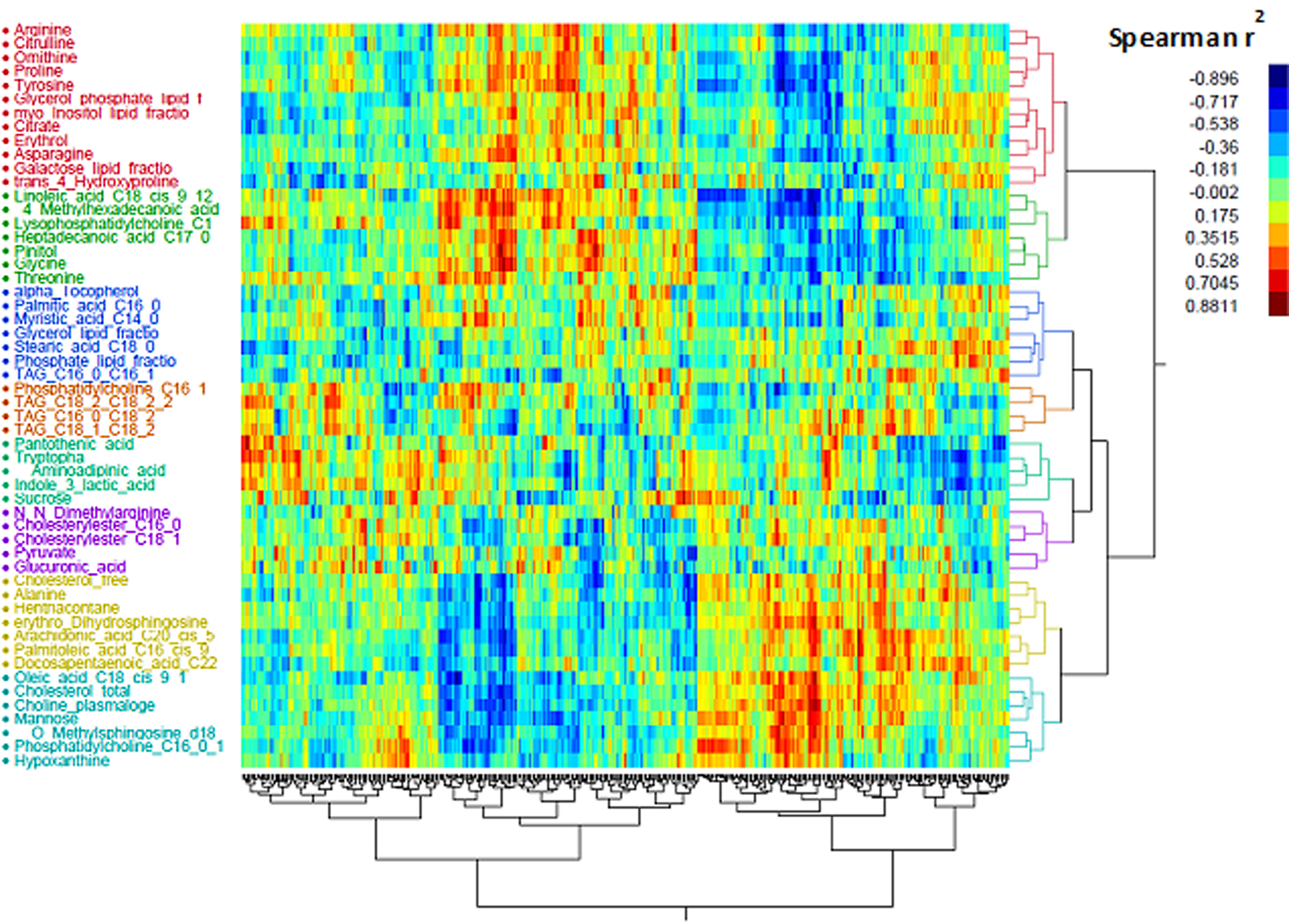

Supplement: S5 Fig — This heat map is similar to the heat map shown in Fig 9 but included all annotated 442 sncRNA and not just the 5-day oscillating sncRNAs. Pairwise metabolite-sncRNA Spearman correlations were generated using quantile-normalized metabolite and sncRNA levels. The range of Spearman correlation values is shown to the right of the figure (brown to blue). The sign of correlation values indicate positive (+) and negative (-) correlation. Clustering within each group (Metabolites or sncRNAs) is based on similarity. (TIF) [file pone.0145919.s005.tif]

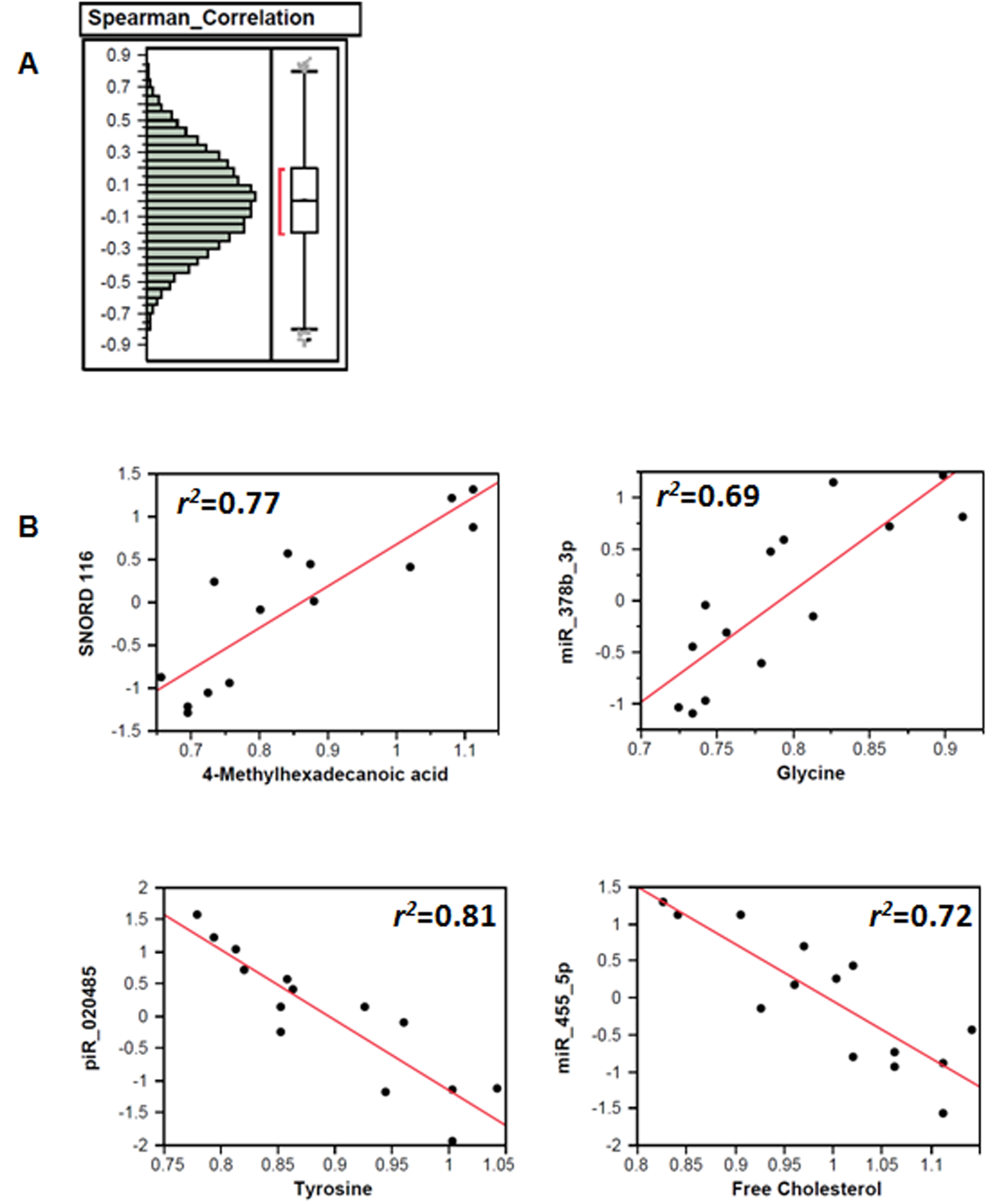

Supplement: S6 Fig — Distribution of 23,632 Spearman correlations corresponding to 422 sncRNA x 56 metabolites (A). Examples of positive and negative sncRNA-metabolite correlations observed in cross-correlation analysis (B). The cross-correlation matrix in S5 Fig is a rich source of metabolome-genome relationships when tests are performed on multidien timescales. For instance, here, the identification of sncRNA mir 455-5p known to inhibit colorectal cancer proliferation and invasion [43] is correlated with free cholesterol levels, and may warrant pharmaceutical research. (TIF) [file pone.0145919.s006.tif]
