# Supplementary material for: The Swine Plasma Metabolome Chronicles "Many Days" Biological Timing and Functions Linked to Growth
Source: PLoS One. 2016 Jan 6;11(1):e0145919. doi: 10.1371/journal.pone.0145919 (PMC4703299; doi:10.1371/journal.pone.0145919)
Supplement: S1 Materials and Methods — Details are provided that address the following points: 1. Animal number; 2. Animals, care, housing, and diets; 3. Indwelling venous catheter placement; 4. Blood collections and plasma separation; 5. Tooth collection, histology, and imaging; 6. Metaboloite profiling; 7. Animal metabolite synchrony; 8. Isolation of plasma small non-coding RNA (sncRNA); 9. Preparation of small RNA-sequencing (sRNA-seq) libraries; 10. Oscillation statistical analysis; 11. Cross-correlation analysis; 12. References. (PDF) [file pone.0145919.s007.pdf]

Supporting Information for

The Swine Plasma Metabolome Chronicles "Many Days" Biological Timing and Functions Linked to Growth

Timothy G. Bromage*, Youssef Idaghdour, Rodrigo S. Lacruz, Thomas D. Crenshaw, Olexandra Ovsiy, Björn Rotter, Klaus Hoffmeier, Friedemann Schrenk

*correspondence to: tim.bromage@nyu.edu

This document includes:

Materials and Methods

Materials and Methods

Animal number

Assuming type 1 and 2 error rates of 5 and 20%, respectively and 14 observations per measure (i.e. one measure/day for two weeks), a correlation between measures within subjects over time of 0.5, assuming 50% non-sphericity, would be satisfied with 18 subjects. This would be sufficient to detect an effect where 25% or more of the variance was attributable to periodicity; we believe that some of the 10% within and 20% between individual variations currently observed in studies of human metabolite profiles are due to the effects of long period rhythms. Cognizant of this, future research is likely reduce this variability.

To provide context, this is considered a moderate effect size in situations where prior data are not available to estimate the potential size of these effects. This estimate is the minimal sample size that is most appropriate for the first sampling strategy.

Animals, care, housing, and diets

Animal care protocols and procedures were reviewed and approved by the University of Wisconsin-Madison Institutional Animal Care and Use Committee (IACUC). The approved protocol number was A01324-0-11-10. Animals were housed in a facility accredited by the Association for the Assessment and Accreditation of Laboratory Animal Care (AAALAC) throughout the experimental procedures.

Thirty-six crossbred (Landrace X Large White X Line 19, ~ 5 mo, 100 kg BW) female pigs (gilts) were divided into 2 groups for replicate trials (n=16 gilts, Trial 1; n=20 gilts, Trial 2). Trial 1 was completed during the period April 24 to May 12, 2011, and Trial 2 during the period of May 15 to June 2, 2012. Gilts were housed individually in pens (2.1 X 0.6 m) that prevented the gilt from turning around to facilitate sample collections. Room temperature and humidity measurements were recorded at 15 minute intervals and mediated throughout the trials. Lights were electronically controlled to provide a 12 h light/dark cycle with lights on at 06:00 and off at 18:00 each day. All emergency and stray-light sources were blocked. Red lights that provided <5 lux light were used to facilitate collections during the dark cycle.

Gilts were fed either a corn-soybean meal control (Ctl) diet or an acidogenic diet (Acd) which was formulated as the Ctl diet plus 2.0% of an anionic salt. The anionic salt is a commercially available product, CAD-MATE, a blend of anionic salts consisting of ammonium sulfate, calcium sulfate, and magnesium chloride (Granco Minerals, Petersburg, VA). Diets were fed for an 18 d trial. Each Trial included a 3-d adjustment period and 14-d collection period. Continuous access to assigned dietary treatments and water were allowed throughout the adjustment and collection periods. Feed consumption was recorded daily.

To identify possible effects of the two diets on metabolite levels, analysis of variance (ANOVA) was performed. The model specification was:

fixed effects: metabolite ~ PERIOD +DAY+DIET+TRIAL
random effect: metabolite ~ 1 | ANIMAL

Overall minor effects were observed below the estimated false positive rate (binominal

test; for $p<0.01$, in total 5 metabolites were significantly changed). In addition, multivariate analysis (Principal component analysis) revealed no group separation of gilts fed with the two different diets.

Three animals acquired an infection attributed to the indwelling venous catheter during the study period and were excluded, rendering a total of 33 animals for metabolite profiling analysis. While a large component of the metabolites independently corroborate the 5-day rhythm, illnesses affected some animals in trial 1, leading to reduced feed intake beginning on day 4.

Indwelling venous catheter placement

A central venous catheter kit (Teleflex ES-04730, 14 ga X 12 in.) was used for placement of an indwelling venous catheter as described by Carroll and colleagues [1]. The placement of indwelling catheters required ~20 min in anesthetized pigs. For anaesthesia a mask was placed on the pig snout and they were sedated initially with 7% sevoflurane and maintained to effect with 2 to 4% isoflurane. The neck region was scrubbed with a surgical disinfectant soap solution. An 18 ga introducer needle was inserted into a brachiocephalic vein. A J-guide wire was inserted into the vein and the needle removed. A tissue expander was then inserted over the guide wire, then the indwelling catheter (14 ga, sterile, polyurethane tubing) was inserted over the wire and the wire removed. The catheter was sutured to skin adjacent to the injection site and taped to the external skin surface. The catheter was protected by a harness vest designed for pigs. Catheters were maintained by daily flushes with 10 mL of a sterile, sodium heparin solution (100 IU/mL) on days between sample collection. On the days of intensive collection catheters were flushed after collection with 10 mL of a sterile, sodium heparin solution (10 IU/mL). Pigs with indwelling catheters were housed in individual stalls to prevent other pigs from dislodging catheters. Catheters were placed 1 to 2 days prior to initiation of the dietary treatments to allow a recovery period before blood collections were initiated. In some animals, catheters that were dislodged were replaced during the trial.

Blood collections and plasma separation

Venous blood was collected (6 mL/sample) at 2 h intervals on day 1, 2, and 14 and once daily on days 3 to 13 using an indwelling venous catheter (4.5 mL volume). Blood samples were collected at 2-h intervals on day 1, day 2 and day 14, beginning at 23:00. On days 3 through 13, blood samples were collected at 23:00 pm each day. For each blood sample collection, a 10 mL syringe was used to withdraw a blood reservoir so that fresh blood was present in the catheter, then a vacutainer tube with K2 EDTA (10.8 mg/tube, BD 367899, 6 mL draw, BD Vacutainer, Franklin Lakes, NJ) was used to collect the retained blood sample. The syringe with a blood reservoir was re-injected into the catheter and flushed with 10 mL of a sterile, sodium heparin solution (10 IU/mL). Within 1 hr from sampling time, the blood sample was centrifuged at 1,500 X g for 10 min at 4°C. The serum separated, and 1.5 mL plasma stored in 2.0 mL Eppendorf tubes flushed with argon gas prior to closure. Plasma samples were immediately frozen in liquid nitrogen. Sample identity was validated and recorded electronically using bar-coded labels on the animal pens, vacutainer tubes, and sample vials, with a time record of the sampling and storage times.

After each trial plasma vials were packed in a water-proof shipping container with dry ice and shipped to Metanomics Health, GmbH, Germany for analysis, taking care to have the dry ice replenished en route and whilst in customs. A total of 1451 samples were subject to metabolite profiling.

Tooth collection, histology, and imaging

Pigs were euthanized prior to tissue collections using procedures described in the 2007 Report of the American Veterinary Medical Association Guidelines on Euthanasia. The pigs were electrically stunned through the brain followed by exsanguination. For each Trial pigs were euthanized on day 15. The lower mandible was dissected from each carcass for histology. A water-cooled saw with a 25.4 cm diamond blade was used to make a vertical cut immediately posterior to the most posterior molar to effectively eliminate the angle of the jaw and ramus. The marrow was rinsed to remove fat and the mandible section was immersed in a 70% ethanol solution. Mandible sections were shipped to NYUCD for histological assessment.

The first permanent molar or fourth deciduous premolar from each experimental animal was dissected free and embedded in polymethylmethacrylate resin, sectioned, ground, polished, and stained for light and electron microscopy examination. Briefly, the tissue was subject to graded ethanol substitution, defatted with acetone, infiltrated with methylmethacrylate resin, and polymerized by exposure to ultraviolet light. The polymerized block was mounted to an EXAKT (EXAKT Technologies, Inc., Oklahoma City, OK) histology slide with cyanoacrylate adhesive, sawn through with the Exakt 300 CP Band System, and ground to ca. 110 μm with the Exakt 400CS Grinding System. The sections were then polished on a Buehler Ecomet III (Lake Bluff, IL) to a 1 μm surface finish and approximately 60-μm section thickness.

Tooth sections were evaluated for their striae of Retzius multidien rhythm. Histological sections were cover-slipped with immersion oil and imaged in conventional light microscopy (LM) and circularly polarized light (CPL) using a Leica-Leitz DMRX/E Universal Microscope using a Leica PL Fluotar 40/0.70 objective lens. LM and CPL images were acquired with a QIClick colour video camera (QImaging, Surrey, BC) using Objective Imaging montaging software (Cambridge, UK). Evaluations of the number of daily increments (cross striations) from two and up to five striae of Retzius repeat periods were obtained in real time and on saved images. If not all daily lines could be observed, averages of cross-striation widths were obtained in local regions and divided into averages of the distance between striae of Retzius, which allowed an estimate of the repeat period; see S1 Table. The enamel of the domestic pig was measured and found to form at a rapid 15-25 micrometers per day. Infradian lines are very common, so to verify the 5-day multidien rhythm from such fields of view and in areas devoid of striae of Retzius, we used non-contact optical interferometry on one specimen to measure the micron height variability due to density variations in the tissue induced as artifact of polishing relief. Daily lines visible between peak ridges along presumptive striae of Retzius confirmed the 15-25 micrometers daily secretion rate and the 5-day multidien rhythm. Measurements on all images were made using a Quantimet QWin (V3.40) image analysis system (Leica Microsystems, Cambridge, UK).

Metabolite profiling

Metabolite profiling was performed at Metanomics Health GmbH, Berlin, Germany. Two types of mass spectrometry analyses were applied to all samples. GC–MS [(gas chromatography-mass spectrometry; Agilent 6890 GC coupled to an Agilent 5973 MS-System, (Agilent, Waldbronn, Germany)] and LC–MS/MS [liquid chromatography-MS/MS; Agilent 1100 HPLC-System (Agilent, Waldbronn, Germany) coupled to an Applied Biosystems API4000 MS/MS-System (Applied Biosystems, Darmstadt, Germany)] were used for broad profiling [2]. Fractionation and derivatisation of samples and detection technologies have been previously described [2-4]. Proteins were removed from plasma samples (60 µL) by precipitation. Subsequently polar and non-polar fractions were separated for both GC–MS and LC–MS/MS analyses by adding water and a mixture of ethanol and dichloromethane. For GC–MS analyses, the non-polar fraction was treated with methanol under acidic conditions to yield the fatty acid methyl esters derived from both free fatty acids and hydrolyzed complex lipids. The polar and non-polar fractions were further derivatised with O-methyl-hydroxyamine hydrochloride (20 mg/ml in pyridine, 50 µL) to convert oxo-groups to O-methyloximes and subsequently with a silylating agent (MSTFA, 50 µL) before GC–MS analysis. For LC–MS/MS analyses, both fractions were reconstituted in appropriate solvent mixtures. High performance LC (HPLC) was performed by gradient elution using methanol/water/formic acid on reversed phase separation columns. Mass spectrometric detection technology was applied as described in the patent US 7196323, which allows targeted and high sensitivity ‘‘Multiple Reaction Monitoring’’ profiling in parallel to a full screen analysis. To account for inter- and intra-instrumental variation in both GC–MS and LC–MS/MS profiling, data were normalized to the median of reference samples derived from a pool formed from aliquots of all samples from that species. Pooled reference samples were run in parallel through the whole process throughout the entire analytical process and used to assess analytical process variability as part of the rigorous quality control performed at the metabolite, sample and experiment (study) level. Data were normalized against the median in the pool reference samples to give pool-normalized ratios (performed for each sample per metabolite). This compensated for inter- and intra-instrumental variation. To classify metabolites at high analytical grade, the limit of detection and the dynamic range of the semi-quantitative measurements were determined by dilution and spiking experiments during method development. Daily, the signal-to-noise (S/N) ratio threshold of 30 was used for a metabolite to be considered ‘‘semi-quantitative’’. Metabolites were annotated at Metanomics Health GmbH by retention times and mass characteristics verified by authentic standards where available or in-depth structure elucidation by carrying out various experiments leading to detailed information of the compound structure. Details on nomenclature are available [3] but in short, the term ‘‘additional’’ was applied to indicate that quantitation can be disturbed by metabolites exhibiting identical analytical characteristics with respect to the quantitation method (S1 Table).

Animal metabolite synchrony

The calculations of population-mean cosinor for any specific metabolite are acquired by measuring the single cosinor relationship for each individual in the sample, and then taking the mean of all fits of all individual wave functions (thus the cosinor statistic does

not require animals to be in phase). Remarkably this procedure on the study sample reveals substantial synchrony among the animals, which among any random sample of individuals with long-period biological rhythms, such as estrous in mammals, would be extremely unlikely for a group of animals in propinquity for the first time. Nevertheless, the 5-day metabolites listed in S5-6 Tables appear to exhibit phasing of individuals as shown in Fig. 5 (main text)

An inspection of the gilt age and breeding data (S1 Table) for the two experimental trials, reveals that the 16 animals of trial 1 were born to 8 sows, while the 20 animals of trial 2 were born to 10 sows, all by artificial insemination with semen derived from one boar. Phasing of acrophase for all animals except #12 (too few data points) is described below and presented in S2 Fig.

Animals from trial 1 were sired and born on the same days. Most animals are in phase, however animal #11 is phase delayed. Interestingly, the acrophase of #11 is also delayed on its 24 hr rhythm, being positioned nearly at the polar opposite of the mean for the population sample. Animals #2 and #6 are phase advanced. compared to most animals of trial 1, though their 24 hr profile is near to the population mean. Because of the uniformity of breeding and birthing schedules in trial 1, taken together, synchrony would appear to be regulated by gestation length or birth, modifiable perhaps by variability in 24 hr rhythms.

Animals from trial 2 were sired and born on a varied schedule. A few animals, #24, #27, and #31, were born on an iteration of seven 5-day cycles later than those of trial 1, and they are in synchrony with the trial 1 animals. Animals #17, #19, and #29 were born to sow 5072, and began the experiment 134 days after birth, at an age equivalent to three 5-day cycles short of trial 1 animals, and they are also in phase with the majority of animals in trial 1. Animals #18 and #33 born to sow 5552 also began the experiment at 134 days, but they were bred one day earlier, and curiously, they are phase delayed by a day (more so #33). Animal #23 born to sow 5762 has the same breeding, birth, and trial schedule as #18 and #33, but it is more or less in phase with trial 1 animals, though at the phase delayed end of the spectrum. Animals #20, #21, and #25 born to sow 5043 were bred on the same day as #17, #19, and #29, but were born two days earlier at 136 days from the beginning of the trial; these animals are phase advanced by two days. Animal #34 is also born on this schedule, but is phase advanced by only a day. Animals #27 and #31 born to sow 5693 are born at 135 days from the beginning of the trial, but rather than advanced by one day, they are in phase with trial 1 animals. Animal #24 also born at 135 days from the trial start as #27 and #31, was bred one day earlier, and curiously again, is phase delayed by a day. Animals #22, #26, #28, and #35 were all born to sow 5722 at 132 days before the trial began, and except for #28 which in phase with trial 1, are phase delayed, but by only about a day instead of two days as might be expected from results of other pigs presented above.

Animal #30 was born at 134 days prior to the trial start, but is advanced by two days. Animals #32 and #36 were born 136 days before trial start, but while #32 was bred one day earlier, it is more or less in phase with trial 1 toward the more advanced end, while #36 is more or less in phase with trial 1 toward the more delayed end.

The sample is too small to enable a formal statement regarding the nature of synchrony in the population sample, but a tendency among many animals suggests that the timing of breeding and births has an effect on phase position. The variability,

however, remains evident even if the data are realigned according to date of birth or gestation length. Variability in phase may be suggested to arise from effects due to some differences existing between sows and gilts, as well as anomalies of 24 hr rhythms. Variability may also be due to errors in recording the date of birth (TD Crenshaw, pers. comm.). Because births sometimes occurred unsupervised at night, the correct date may have been misidentified. It is also possible that births at night have an effect on phase relationships, swinging the phase one way or the other depending upon the time in the 24 hr cycle, or some as yet unidentified differences between sows and/or newborns.

Isolation of plasma small non-coding RNA (sncRNA)

Plasma sncRNA was extracted using the exoRNeasy kit (Qiagen) as described by the manufacturer with some minor modifications. Because the remaining plasma sample volumes were small, they were pooled from six animals to provide volumes large enough at each time period to perform the extractions. We chose three animals from each of the two trials to include animal numbers 1, 7, 10, 17, 27, and 30. The samples were then centrifuged 10 min at 16000g and the supernatant was filtered using 0.8 µm membrane (Millex-AA, Millipore). 4 ml plasma were mixed with one volume XBP buffer and loaded on the exoEasy spin column for extraction.

Extracts were washed with 10 ml XWP buffer and lysed with 700 µl QIAzol. 90 µl chloroform were added for subsequent phase separation. One volume of ethanol (70%) was added to the aqueous phase and loaded on the RNeasy MinElute spin column. One volume of ethanol (100%) was added to the flow-thru and loaded on a new RNeasy MinElute spin column, washed with 500 µl RPE buffer and 500 µl ethanol (80%), and eluted with 15 µl water (two times). RNA was quantified using a Qubit Fluorometer (Invitrogen).

Preparation of small RNA-sequencing (sRNA-seq) libraries

For preparation of small RNA libraries, 20 ng RNA (small RNA fraction) was spiked with 1 fmol cel-miR-39 RNA, ligated (NEBNext Small RNA Library Prep, NEB) to modified 3' and 5' adapters (TrueQuant RNA adaptors, GenXPro). Adapter-ligated RNA was reverse transcribed (First-Strand Synthesis System, Life Technologies), purified with SPRI beads (SPRIselect, Beckman Coulter), PCR-amplified with 16 cycles (KAPA HiFi Hot-Start Polymerase, KAPA Biosystems), purified (Amicon Utracel-10, Millipore), and size-selected by polyacrylamide gel electrophoresis. The small RNA library was sequenced on an Illumina HiSeq2000 machine with 1x50 bps.

The number of unique sncRNA sequences recorded equals 47762 (422 annotated to the pig genome), and among these, 38803 had sufficient data to perform cosinor analysis, from which 1695 were found to have a statistically significant 5-day rhythm ($p < 0.05$). Despite this, the concentration values for many of these 5-day sncRNAs are low, and thus we arranged the full data set by mean concentration (from highest to lowest) and arbitrarily examined the top 3000 small molecules (i.e., those having the highest concentration). Among these, 70 sncRNAs had a statistically significant 5-day rhythm. That these sequences have the shortest length in most cases (S10 Table), suggests that only mature sncRNA are typically oscillatory, and that premature sequences are typically too transient to be expressed rhythmically; in only one case a non-significant mature

sncRNA was accompanied by a precursor with a statistically significant 5-day rhythm (TCACCGGGTGTAATCAGCTG).

All concentration (Ct) values are relative. To each sample we spiked-in the same amount of an external DNA-Fragment from *Xenopus laevis*. In the qPCR we measured the Ct values of both the *Sus scrofa* DNA and the Xenopus laevis DNA. Then we calculated the difference (ΔCt) of both and normalized it (ΔΔCt) against the 1st sample (day 1 at 23:00 h). Thus the quantity of all samples are relative to the 1st sample.

Oscillation Statistical analysis

The cosinor method described here was selected for the evaluation of time series data [5,6]. The cosinor was designed to test for *anticipated* periodicities (i.e., our 5-day and 10-day periods in this study). This means that if we expect a priori to find, say, a 5-day multidien rhythm, we can fit a 5-day cosine curve to the data, and if the *p*-value is less than 0.05, it can be concluded that the multidien rhythm was demonstrated. The cosinor would not be used if none of the components was anticipated, in which case it would be customary to compute a spectrum in a range of frequencies to determine which components may lie above the noise level.

Some believe that the cosinor works in the time domain by contrast to a Fourier analysis. For equidistant data covering an integer number of cycles, the least squares spectrum obtained by cosinor (at the Fourier frequencies) is *identical* to the Fourier transform; it is not a matter of technique. The problems associated with testing for periodicities are more or less the same whether data are analyzed by cosinor or by other spectral analysis techniques. Attempts to analyze our data by unsupervised spectral techniques, however, failed, because these techniques are sensitive to the number of available cycles, requiring at least 3 compared to our less than 3 cycles. However, we did subject all 55 metabolites oscillating on the 5-day period length and all 11 metabolites oscillating on the 10-day period length to population-cosinor tests of all period lengths from 2-10. These metabolites only achieved highest statistical power for their respective periods.

We introduced the cosinor equations into Matlab R2013a (The Mathworks, Inc. Natick, MA) for semiautomated processing. Statistics retrieved include Mesor, Amplitude, Acrophase, p-value, and percent rhythm (described below).

I. Single Cosinor (employed for the time series analysis of a single animal):

The cosine function is used as a model for the evaluation of biological rhythms:

(1) $y_j = M + A\cos(\omega t_j + \varphi)$

with the y_j value at time t_j for j = 1,2…n measurement. The following parameters of the function were defined as follows: Mesor (M) is the value about which oscillations occur, Amplitude (A) is half the difference between the highest and the lowest values, angular frequency (ω) is degrees/unit time, with 360º representing a complete cycle, and

acrophase (φ) is time in degrees at which the first peak of the rhythm occurs. The equation (1) in linear form is:

$$(2)\ y_j = M + \beta x_j + \gamma z_j$$

where $x_j = \cos(\omega t_j)$, $z_j = \sin(\omega t_j)$, $\beta = A\cos(\varphi)$, and $\gamma = -A\sin(\varphi)$. The known parameters are x_j and z_j with the frequency being computed as $\omega = 2\pi/P$, where P is the given period length in hours. The unknown parameters M, β, and γ were obtained by solving the system of normal equations (NE) simultaneously:

$$(3)\ \text{NE} = \begin{bmatrix} n & \sum_{j=1}^{n}(x_j) & \sum_{j=1}^{n}(z_j) & \sum_{j=1}^{n}(y_j) \\ \sum_{j=1}^{n}(x_j) & \sum_{j=1}^{n}(x_j^2) & \sum_{j=1}^{n}(x_j z_j) & \sum_{j=1}^{n}(x_j y_j) \\ \sum_{j=1}^{n}(z_j) & \sum_{j=1}^{n}(x_j z_j) & \sum_{j=1}^{n}(z_j^2) & \sum_{j=1}^{n}(z_j y_j) \end{bmatrix}$$

Knowing β and γ, the amplitude and acrophase in radians could be obtained by the following equations:

$$(4)\ A = \sqrt{\beta^2 + \gamma^2} \quad (5)\ \theta = \tan^{-1}\left(\left|\frac{\gamma}{\beta}\right|\right)$$

In order to convert the acrophase from radians to degrees, the quadrant for θ is determined:

$$(6)\begin{cases} if\ \beta > 0\ and\ \gamma = 0\ or\ \gamma < 0, \varphi = -2\pi + \theta \\ if\ \beta < 0\ and\ \gamma > 0\ or\ \gamma = 0, \varphi = -\pi + \theta \\ if\ \gamma > 0\ and\ \beta > 0\ or\ \beta = 0, \varphi = -\theta \\ if\ \gamma < 0\ and\ \beta < 0\ or\ \beta = 0, \varphi = -\pi - \theta \end{cases}$$

A zero-amplitude test was performed to calculate the probability of the amplitude (A) being significantly different from zero. In this test, the F-distribution was used with degrees of freedom ν1 = 2, ν2 = n-3, and α = 0.05. The p-value was obtained by solving the equation below (the right hand side of this equation is a Matlab representation of the F-distribution):

$$(7)\ p\text{-value} = 1 - F\left(x = \frac{MSS}{2} \Big/ \frac{RSS}{n-3} \,\middle|\, v1, v2\right) = \int_0^x \frac{\Gamma\left(\frac{v1+v2}{2}\right)}{\Gamma\left(\frac{v1}{2}\right)\Gamma\left(\frac{v2}{2}\right)} \left(\frac{v1}{v2}\right)^{\frac{v1}{2}} \frac{t^{\frac{v1-2}{2}}}{\left[1+\left(\frac{v1}{v2}\right)t\right]^{\frac{v1+v2}{2}}} dt$$

where MSS is the model sum of squares and RSS is the residual sum of squares that are represented by the equations:

(8) $RSS = \sum (y_j - (M + \beta x_j + \gamma z_j))^2$

(9) $MSS = \sum ((M + \beta x + \gamma z) - \bar{y})^2$, where $\bar{y}$ is the mean of y values.

In addition to the p-value, the percent rhythm (PR) was estimated in order to see how well the cosine model fits the observed values in the time series. In other words, PR represents the total variance accounted for by the fitted cosine wave.

(10) $PR = \dfrac{MSS}{MSS + RSS} \times 100\%$

II. Population-mean cosinor (employed for the time series analysis of a sample of animals):

The same cosine model (1) was used for the evaluation of the biologic rhythmicity in a population with a given period length. Parameters M_i, β_i and γ_i were obtained with the equation (3) for the population i = 1,2,…k, where k is a total number of animals. The sample means are $\overline{M}, \bar{\beta}$ and $\bar{\gamma}$:

(11) $\overline{M} = \dfrac{1}{k}\sum_{i=1}^{k} M_i$ (12) $\bar{\beta} = \dfrac{1}{k}\sum_{i=1}^{k} \beta_i$ (13) $\bar{\gamma} = \dfrac{1}{k}\sum_{i=1}^{k} \gamma_i$

The mean amplitude ($\bar{A}$) and mean acrophase ($\bar{\Phi}$), where $\bar{\Phi}$ was converted to degrees using one of the equations in (6), have the following calculations:

(14) $\bar{A} = \sqrt{\bar{\gamma}^2 + \bar{\beta}^2}$ (15) $\bar{\Phi} = \arctan\left(\left|\dfrac{\bar{\gamma}}{\bar{\beta}}\right|\right)$

The cumulative probability density function, F-distribution, in the population-mean cosinor model is solved for the p-value differently from the cumulative probability density function in a single cosinor. The degrees of freedom ν1 and ν2 were equal to 2 and k-2, respectively, and α error was equal to 0.05. A solution for the p-value is as follows:

(15) $p\text{-value} = 1 - F\left(\left(\dfrac{k(k-2)}{2(1-R^2)(k-1)}\right)\left[\dfrac{\bar{\beta}^2}{\sigma_\beta^2} + \dfrac{2R\bar{\beta}\bar{\gamma}}{\sigma_\beta \sigma_\gamma} + \dfrac{\bar{\gamma}^2}{\sigma_\gamma^2}\right] \middle| v1, v2\right)$

where σ_β^2 and σ_γ^2 are the variances of β and γ, $\sigma_{\beta\gamma}$ is the covariance of β,γ, and r is a correlation coefficient:

(16) $\sigma_\beta^2 = \dfrac{1}{k-1}\sum_{i=1}^{k}(\beta_i - \bar{\beta})^2$ (18) $\sigma_{\beta\gamma} = \dfrac{1}{k-1}\sum_{i=1}^{k}(\beta_i - \bar{\beta})(\gamma_i - \bar{\gamma})$

(17) $\sigma_\gamma^2 = \dfrac{1}{k-1}\sum_{i=1}^{k}(\gamma_i - \bar{\gamma})^2$ (19) $R = \dfrac{\sigma_{\beta\gamma}}{\sqrt{\sigma_\beta^2 \sigma_\gamma^2}}$

The PR for the population-mean cosinor was calculated by:

(20) $$\overline{PR} = \frac{\overline{MSS}}{\overline{MSS} + \overline{RSS}}$$

where MSS and RSS are the mean model sum of squares and mean residual sum of squares calculated by:

(21) $$\overline{RSS} = \frac{1}{k}\sum_{i=1}^{k} RSS_i$$ (22) $$\overline{MSS} = \frac{1}{k}\sum_{i=1}^{k} MSS_i$$

Cross-correlation analysis

Cross-correlation analysis was done by calculating pairwise metabolite-sncRNA Spearman correlations using quantile-normalized metabolite and sncRNA levels. sncRNA levels for 1-14 days were generated using small RNA-sequencing of a pool of plasma samples from six animals as detailed above. Metabolite levels for 1-14 days used in this analysis correspond to the mean value across the same set of six animals for each metabolite. Quantile normalization of sncRNA and metabolites and hierarchical clustering of the correlation matrix was done using the Ward method as implemented in JMP Genomics (SAS Institute, Cary NC, USA).

References

1. Carroll JA, Daniel JA, Keisler DH, Matteri RL (1999) Non-surgical catheterization of the jugular vein In young pigs. Lab Animal 33: 129-134.
2. Ravenzwaaay Bv, Cunha GC, Leibold E, Looser R, Mellert W, et al. (2007) The use of metabolomics for the discovery of new biomarkers of effect. Toxicology Letters 172: 21–28.
3. Saito RI, Smoot ME, Ono K, Ruscheinski J, Wang PL, et al. (2012) A travel guide to Cytoscape plugins. Nature Methods 9: 1069-1076.
4. Roessner U, Wagner C, Kopka J, Trethewey RN, Willmitzer L (2000) Simultaneous analysis of metabolites in potato tuber by gas chromatography-mass spectrometry. Plant Journal 23: 131–142.
5. Bingham C, Arbogast B, Guillaume GC, Lee J-K, Halberg F (1982) Inferential statistical methods for estimating and comparing cosinor parameters. Chronobiologia 9: 397-439.
6. Nelson W, Tong YL, J.-K.Lee, Halberg F (1979) Methods for cosinor-rhythmometry. Chronobiologia 6: 305-322.
7. Chai J, S.Wang, Han D, Dong W, Xie C, et al. (2015) MicroRNA-455 inhibits proliferation and invasion of colorectal cancer by targeting RAF proto-oncogene serine/threonine-protein kinase. Tumor Biology 36: 1313-1321.
